# A facile photonics reconfigurable memristor with dynamically allocated neurons and synapses functions

**DOI:** 10.1038/s41377-025-01928-5

**Published:** 2025-08-12

**Authors:** Zhenyu Zhou, Lulu Wang, Gongjie Liu, Yuchen Li, Zhiyuan Guan, Zixuan Zhang, Pengfei Li, Yifei Pei, Jianhui Zhao, Jiameng Sun, Yahong Wang, Yiduo Shao, Xiaobing Yan

**Affiliations:** 1 Key Laboratory of Brain-Like Neuromorphic Devices and Systems of Hebei Province, College of Electron and Information Engineering, School of Life Sciences, Institute of Life Science and Green Development, Hebei University, Baoding, China; 2 School of Electronic Science and Engineering, Xiamen University, Xiamen, 361005 China

**Keywords:** Optical techniques, Optics and photonics

## Abstract

The dynamic neural network function realized by reconfigurable memristors to implement artificial neurons and synapses is an effective method to complete the next generation of neuromorphic computing. However, due to the limitation of reconfiguration conditions, there are inconsistencies in the turn-on voltage and operating current before and after the reconfiguration of neuromorphic devices, which leads to huge difficulties in hardware application development and is an urgent problem to be solved. In this work, we introduced light as a regulatory means in the memristor and achieved the reconfiguration of volatile (endurance ~10^6^ cycles) and non-volatile (retention ~10^4^ s) characteristics with a unified working parameter through the photoelectric coupling mode. The switching voltage of the device can be controlled 100% by this method without any limiting current. This will allow neurons and synapses to be dynamically allocated on demand. We completed the verification such as Morse code decoding, Poisson coded image recognition, denoising in the image recognition process, and intelligent traffic signal recognition hardware system under different work modes. It is verified that the device can dynamically adjust the neuromorphic according to needs, providing a new idea for the further integration of neuromorphic computing in the future.

## Abstract (web summary)

Photonics reconfigurable memristor simulate volatile and non-volatile properties to achieve dynamic modulation of neurons and synapses and demonstrate application scenarios.

## Graphical abstract

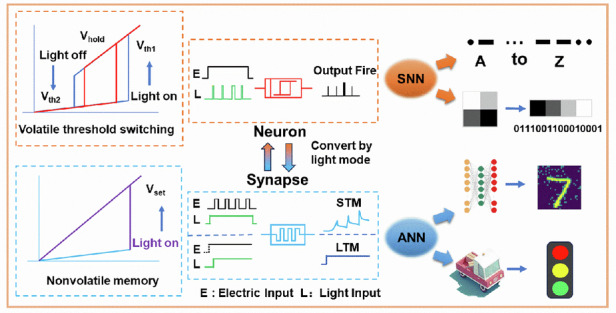

## Introduction

Artificial intelligence field has huge challenges in continuous learning, mainly because when new data increments are presented to neural networks, they will interfere with the previously learned knowledge and fail to complete the continuous learning task, leading to catastrophic forgetting^1,2^. It is found that achieving effective resource allocation through dynamic networks and thereby enabling compatibility among multiple neural network computations is one of the main approaches to addressing this issue^3^. In biology, there is no dynamic distribution process between neurons and synapses. Neurons are responsible for transmitting nerve signals and information processing, and synapses are the connection points between neurons that transmit signals^4^. Their functions and structures are highly characterized and specialized in living organisms to perform complex nervous system functions. Memristors have been extensively studied in the field of neuromorphic computationally^5–8^. Biomimetic neurons and synapses that use memristors to implement pulsed neural networks and artificial neural networks are also becoming more mature^9^. At present, researchers mainly optimize a certain function to meet the configuration requirements of the target neural network, such as pulse neural network or artificial neural network, so the existing optimization methods hinder the realization of dynamic neural network^4^. The function of volatile integration activation and non-volatile synaptic weight regulation is the basic unit of neuromorphic computational network, and it is also the necessary condition for constructing dynamic network to realize continuous learn^9^. The reconfigurable memristor behavior with volatile and non-volatile has been observed^4,9–11^, which demonstrates the ability to achieve dynamic distribution of neurons and synapses in a single device. However, due to the limitations of material and mechanism, the reconstructed device’s working voltage, high resistance state (HRS), low resistance state (LRS), and operating current cannot be maintained consistently, which significantly increases the difficulty of hardware application development and impedes its further progress in dynamic neural networks^4,9–11^. Therefore, the development of novel reconfigurable memristors to realize the dynamic neural network function of artificial neurons and synapses is an effective way to complete the next generation of neuromorphic computation^9^.

In recent years, neuromorphic computing based on photoelectric memristors has overcome the limitations of traditional von Neumann architecture, and it has been extensively studied in the field of visual perception systems integrating sense, memory and computation^12–17^. Since light can pass through three-dimensional space without interfering with each other, and light has advantages such as wide broadband, easy parallel input, and good scalability^18,19^, these unique optical properties can perform neuromorphic functions at high speed. Therefore, light as an input port of information, can greatly enrich the freedom of synaptic plasticity regulation^20,21^. In addition, light can greatly increase the electron-hole pair concentration of the device in a short time, and increase the device current through the separation of the barrier under the action of the electric field^21^, so that the device changes from a high resistance state to a low resistance state. And because of the photogenerated carriers, the device does not require the traditional vacancy or ion conduction filament. More importantly, it does not need electroforming or limiting current to assist it to achieve the corresponding working state, so it will not destroy the internal crystal structure of the device, which helps to improve the stability and uniform of the device when working, and it may achieve device-to-device consistency without calibration in neuromorphic computing, which is an extremely important property^22^. Therefore, the introduced light is expected to provide a promising way to dynamically allocate resources and greatly promote the development of new in-memory computing technologies. It provides a new solution for neural computing system based on dynamic allocation.

In this paper, we design photonics reconstructed memristor (PRM) based on the simple structure of Pd/ZnO/Graphene/SiO_2_/Si. We find that changing the photoelectric coupling mode can control the changes of the volatile and non-volatile characteristics of the device, under the premise of ensuring the high unity of operating voltage, high and low resistance state and operating current of the device, we construct an artificial neuron and synaptic model device with dynamic distribution capability. In neuron mode, the device exhibits a stable and controllable threshold feature, and it can convert different frequencies of light into corresponding spike pulses, which is the basic feature of the excitation function of simulated neurons, and can be used to realize the decoding of Morse cipher information based on pulse code and Poisson coded image recognition. In the synaptic mode, the device exhibits non-volatile memristor characteristics, which can simulate two plasticity states of short-term memory (STM) and long-term memory (LTM). In the STM mode, the neural network constructed by the EPSC effect generated by the photoelectric coupling realizes MNIST handwritten recognition. In the LTM mode, an intelligent driving hardware system based on LTM is designed to recognize traffic lights repeatedly. The four scenarios fully prove that the PRM can integrate the functions of artificial neurons and synapses through a single device, and thanks to the high consistency of the electrical parameters of the device, the application circuit is greatly simplified, and it is a new idea for realizing high-speed, high-density and reconfigurable brain-like computing.

## Results

### Reconfigurable photoelectric memristor

**Fig. 1:**
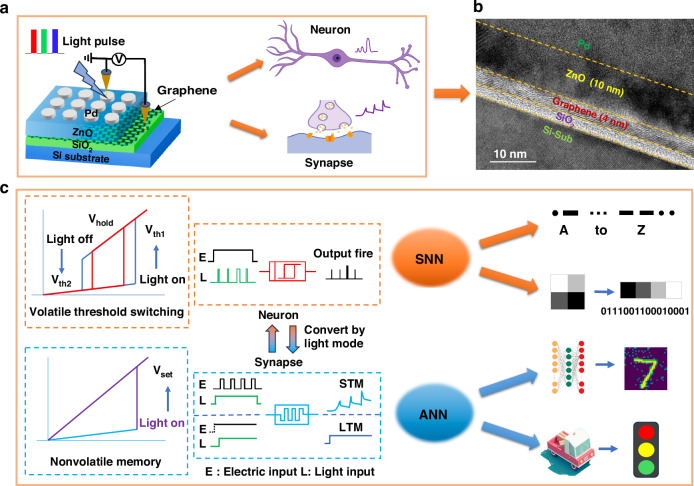
**Schematic of optically PRM**. **a** Schematic image of device structure, the light pulse indicates that the device can achieve the perception of RGB tricolor light. **b** HRTEM image of the Pd/ZnO/Graphene/SiO_2_/Si device, and the ZnO is a polycrystalline film (red and green boxes) with a thickness of 10 nm, and the graphene is a multilayer film with a thickness of 4 nm. **c** Reversible volatile threshold-switching memristor (VTSM) and nonvolatile memristor (NVM) in PRM, where both neurons and synapses are reversible transformations achieved by changing light mode, and the two models are used to study the spiking neural networks (SNN) and artificial neural networks (ANN)

To construct a dynamic neural network, we design a reconfigurable photoelectric memristor (PRM) device. Its structure is Pd/ZnO/Graphene/SiO_2_/Si (Fig. 1a and see more details in Method), in which the multilayer graphene with excellent electrical conductivity is the positive electrode and Pd is the negative electrode. And it takes advantage of the programmability of light to realize the properties of neurons and synapses in a single device. By analyzing the cross-sectional structure of high-resolution TEM images of the device, it is found that the device has a simple sandwich structure, and the longitudinal size of the device is small: the thickness of the graphene electrode is only and 4 nm thickness of the ZnO layer is 10 nm and the ZnO film contains oxygen vacancies (Fig. 1b and Fig. S1, Supplementary Information). The structure uses photogenerated charge carriers to increase the device current and realize fast switching of high resistance states (HRS) and low resistance states (LRS) under the combined action of electric field and illumination, which can be turned on for up to 9.48 μs and turned off for up to 24.16 μs (Fig. S2, Supplementary Information). Moreover, since the device can be turned on by adding light and turned off by removing light, the switch of the device is controlled by light to simulate the characteristics of the volatile threshold-switching memristor (VTSM) and nonvolatile memristor (NVM), and ultimately achieve the performance of neurons and synapses (Fig. 1c), to meet the dynamic configuration requirements of specific functions of neuromorphic devices in different environments^3^.

**Fig. 2:**
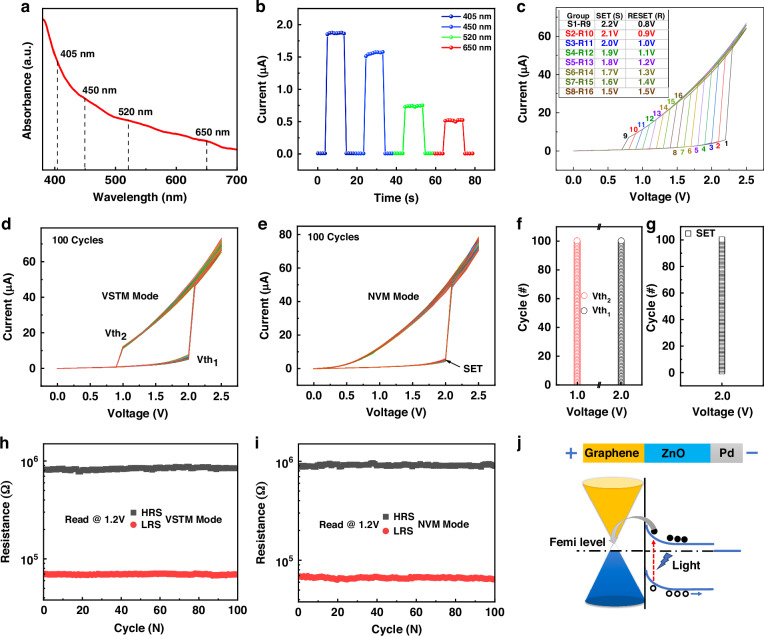
**Basic optical response characteristics of PRM**. **a** Ultraviolet-visible absorption spectra of device. **b** The response of the device to light at different wavelengths. **c** Different threshold switching voltages of devices under optical control. SET electrical compression is written as “S”, and Reset voltage is abbreviated as “R”, where S1-R9 represents one set of I-V curves S1 is the on voltage, and R9 is the corresponding off voltage. **d** The I–V curve for 100 cycles under VSTM Model. **e** The I-V curve for 100 cycles under NVM Model. **f** The distribution of 100 groups of switching voltages (Vth_1_ and Vth_2_) extracted from (**d**). **g** The distribution of 100 groups of SET voltages (Vth_1_ and Vth_2_) extracted from (**d**). **h** The distribution of 100 groups of HRS and LRS extracted from (**d**). **i** The distribution of 100 groups of HRS and LRS extracted from (**e**). **j** Energy band diagram of the graphene/ZnO under light irradiation

Neurons and synapses are the building blocks of brain-like systems, and memristors with highly uniform switching characteristics are needed to effectively construct artificial neural networks^23^. The PRM devices provide the ability to program electronic circuits on demand to dynamically implement neuronal and neurosynaptic features. According to the light absorption diagram (Fig. 2a) and the optical response of the device (Fig. 2b and Fig. S3–S5, Supplementary Information) the device can respond to the light at different wavelengths and powers, and can maintain more than 10^4^s stability under continuous light, which lays a solid foundation for the following dynamic light regulation. First, we use light with a lower energy density (520 nm, 50 mW, 0.228 nW/μm^2^) to regulate the threshold mode of the device (Table S1, Supplementary Information). In the process of 0 V ~ 2.5 V ~ 0 V voltage scanning, the device is illuminated at 2.2 V, and the device is quickly turned on from the high resistance state to the low resistance state until the light is removed at 0.8 V, so that the device returns to the high resistance state. The I–V curve change of the threshold mode was completed (See Fig. 2c). We measured the I-V curves of 8 groups of different switching voltages, where S1–S8 and R9–R16 correspond to the SET voltage and RESET voltage of each group respectively (Fig. 2c). The threshold switching state achieved by the programming mode of light does not require any limiting current, and can be switched at any voltage value (Figs. S6 and S7, Supplementary Information). It is fully explained that the PRM device can adjust the switching voltage on demand to adapt to different hardware circuits without limiting the current. This is not implemented in the same way as other threshold devices (Table S2, Supplementary Information). In addition, the optically implemented VSTM mode are achieving 100 cycles I–V curve at an on voltage (Vth_1_) of 2.0 V and an off voltage (Vth_2_) of 1.0 V very stalely (Fig. 2d), and it can implement the DC mode of the memristor (Fig. S8, Supplementary Information), for comparison, we only tested 100 cycles of forward I–V of stable VNM mode (Fig. 2e). Indicating that the switching voltage (Vth_1_ and Vth_2_ in VSTM mode or SET in VNM mode) is extremely uniform and controllable (Fig. 2f, g). And we also extracted the HRS and LRS resistance values of I–V curves at 1.2 V under VTSM and NVM mode respectively (Fig. 2h, i), those switching resistance did not obvious difference, indicating that the resistance values of the HRS and LRS of the device can be highly consistent when the two modes are switched, which also fully proves that the light-controlled threshold phenomenon has great advantages. To further verify its stability, we tested the device to device (Fig. S9, Supplementary Information) and also simulated the threshold switching for 10^6^ times in the light pulse and electrical pulse modes. After a certain number of pulse switches, the I–V curve is measured once, obtaining the I-V curve with different pulse times (Fig. S10, Supplementary Information), which lays the foundation for building neurons and synapses^3,23^.The difference between synapses and neurons is that synapses have plasticity, non-volatility is the most important characteristic of simulated synapses^24^. Fortunately, the PRM devices can easily achieve two neuromorphic transformations by means of light regulation. The reason is that the conductive behavior of the device does not depend on the conductive filaments (Fig. S11, Supplementary Information), whether at high DC voltage or pulse voltage, or at small read voltage, the resistance state of the device can be controlled by an optical switch. This form of regulation is quite different from the previous behavior of regulating high and low resistance states by relying on conductive filaments^25^. Figure 2j is a schematic diagram of Pd/ZnO/Graphene energy bands under light irradiation. Electron-hole pairs generated at the optical irradiation interface increase the free carrier concentration, under the action of electric field, electrons are transferred to the graphene layer to form an electron flow^26,27^, and holes are transferred to the Pd electrode layer to form a hole current. Therefore, a large photocurrent and dark current ratio is achieved under the action of light, which lays a foundation for realizing the reconfigurable memristor.

### Encrypted information communication at RPM-VTSM mode

**Fig. 3:**
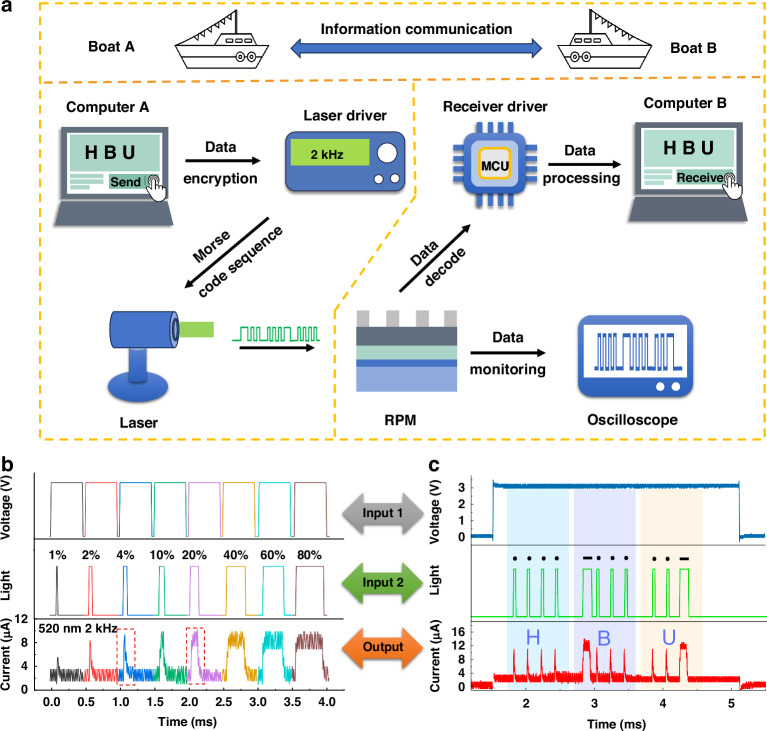
**Light pulse signal and data transmission decoding based on Morse code**. **a** Schematic of data encryption transmission and decryption. **b** The current response of the PRM device under same electrical pulse and different light pulse duty cycle. Here 4% and 20% of the 2 kHz illumination were selected to simulate the short and long signal states for further testing. **c** Single PRM device data decoding of the “HBU” Morse code

The PRM device can be used in scenarios that rely on light as the medium of information transmission. Taking navigation as an example in the vast sea, lamp signal plays a very important role in the information exchange of navigation, which can carry out reliable communication in long distance and harsh sea environment. Ships can transmit important navigational information to each other through sea lights, such as heading, speed, distance, etc^28,29^. The PRM device can decode the navigation information in real time and quickly, which is essential for communication and coordination between ships. The ship encrypts the transmitted signal with the encoding method of Morse code through the computer A, and controls the switching state of the laser diode to transmit the signal. Then, another ship computer B receives the signal through the PRM and produces the corresponding short photocurrent point signal “.” and long photocurrent signal “—”, which is eventually restored by the driver to a human-recognizable signal (Fig. 3a). Under the combined action of an electric field and a light pulse with a variable duty cycle or frequency, the PRM device will also produce an optical response of different intensity and different time scales (Fig. 3b), which means that the device can adjust it conduction state and conduction time by changing the input mode of light, to achieve a variety of forms of control (see more details in Fig. S12–S14, Supplementary Information). We selected 2 kHz light pulses with a duty cycle of 4% and 20% to simulate short point signal “.” and long signal “—”in the Morse code to compile the encrypted message, and it successfully emulates the Morse code A through Z and the 0–9 numeric symbols (see more details in Figs. S15 and S16, Supplementary Information). To simulate the information transmission in the navigation process, we encode the three letters “H-B-U” in the form of Morse code, and then transmit the encoded information to the optical driver module to control the transmission of the encoded light pulse, and receive the information and decode it after the PRM device (Fig. 3c). The PRM device quickly and accurately decodes the received information into the Morse code corresponding to “H-B-U” under the combined action of the 2 V voltage and the light pulse train carrying the information. Experiments show that a single PRM device can realize the function of encryption transmission and decoding based on optical information, which is low cost, fast and convenient, and more conducive to navigation safety.

### Image recognition at PRM-VTSM mode

**Fig. 4:**
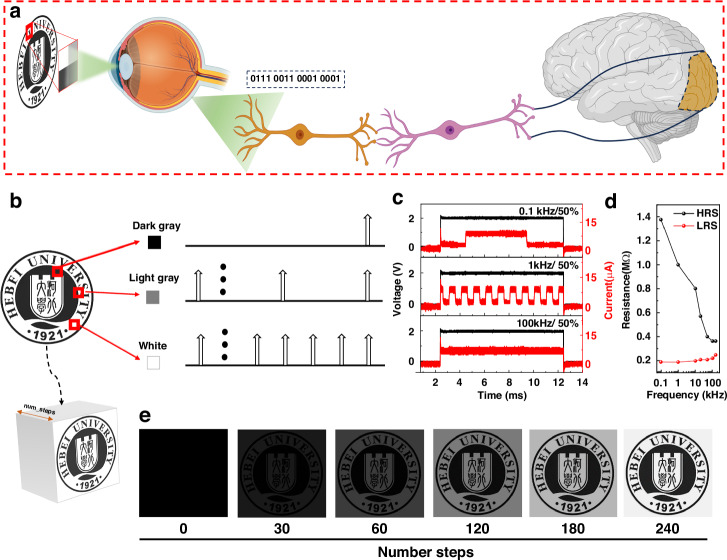
**Simulating visual perception neuron image recognition with VTSM mode**. **a** The process diagram of images being converted into Poisson encoding and then transmitted as a sequence of pulses to the brain. **b** Poisson encoding for different pixel values encoding frequency and time scale relationship. In image recognition, the image needs to be accumulated on time step scale. The pulse train is represented on a time scale by number steps. **c** PRM device response at 0.1 kHz, 1 kHz, and 100 kHz light pulses. **d** The relationship between HRS and LRS resistance values of PRM devices and different optical frequencies. **e** Poisson encodes the cumulative display of image information at different number steps

Poisson coding is a coding technique utilized in SNN, incorporating the statistical principles of the Poisson distribution^30^. Unlike traditional frequency coding methods, Poisson coding associates a neuron’s firing probability with the stimulus intensity, allowing for sparse neuronal firing to encode information. This approach represents the gray-level information of an image by linking the probability of neuron firing to pixel brightness levels. The image information is converted into Poisson code by the device and transmitted to the brain (Fig. 4a). Neurons with higher firing probabilities (associated with brighter pixels) emit pulses more frequently, while those with lower firing probabilities (associated with darker pixels) emit fewer pulses (Fig. 4b). This method takes advantage of the natural tendency of neurons to fire in bursts, making it a more effective and biologically plausible encoding strategy. Notably, Poisson coding has garnered attention in neuroscience and impulsive neural network research due to its efficiency and compatibility with biological systems. The more times the Poisson coding is superimposed on the time line, the more accurate the brightness information of the pixels, and the higher and clearer the recovery degree of the obtained image. PRM device can achieve conductivity regulation at different frequencies by photoelectric hybrid mode as shown in Fig. 4c, d and Supplementary Information Fig. S17. The device completes a conversion of high and low resistance states under the light pulse of 0.1 kHz and 50% duty cycle, and the high resistance state can reach about 1.4 MΩ. Under the same electric pulse, when the light frequency gradually increases, the device shows a higher frequency of high and low resistance states, and the resistance value of the high resistance state gradually decreases with the increase of frequency. The value of the conductance is adjusted according to the frequency of the light pulse. Figure 4e shows the image recognition process after the simulated image is sent to the device in neuron mode through Poisson coding. It can be clearly seen that the image is completely black when the pulse is sent for 0 or very few pulses. With the increase of superposition times, the pulse frequency of corresponding pixels increases, and the image is gradually clear, and the image recognition is finally completed.

### Image recognition at RPM-NVM mode

**Fig. 5:**
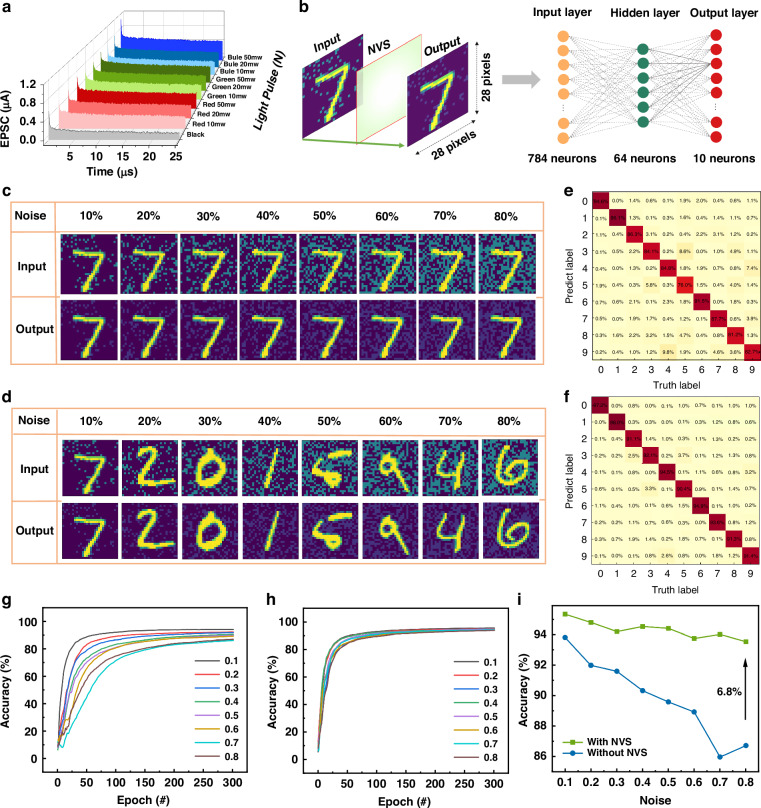
**Simulation of ANN for image recognition by the PRM photoelectric synapse**. **a** The EPSC of PRM photoelectric synapse. **b** Schematic illustration of the simulated ANN with a three-layer architecture. **c** Numeral images 7 before and after pre-processing under various noise levels. **d** Random numeral images before and after pre-processing under various noise levels. **e** Confusion matrix for recognition of numeral images-7 after NVS 300 epochs training. **f** Confusion matrix for recognition of random digital images after NVS 300 epochs training. **g** The relationship between training times and recognition accuracy under different noises without NVS processing. **h** The relationship between training times and recognition accuracy under different noises with NVS processing. **i** Comparison of different noise recognition accuracy with or without NVS

Photoelectric synapses play an important role in image pre-processing based on neuromorphic devices^31,32^. Traditional image sensors inevitably produce noise in images due to thermal noise from electronic components and readout errors during operation^33,34^. Noise removal methods usually use smoothing and filtering algorithms to preserve image details and structural information while reducing the impact of noise on image quality, but this will increase the corresponding cost^35^. To solve this problem, we use PRM devices to construct a neuromorphic vision sensor (NVS) based on STM synaptic properties in NVM mode to simulate the noise removal process of image recognition. Photoelectric synapses have EPSC plasticity at STM model shown in Fig. 5a and Supplementary Information Figs. S18, S19. In the process of image perception, EPSC neuromorphic characteristics of PRM devices under photoelectric action are utilized to effectively remove noise in images^36^. To better demonstrate the ability of NVS to remove noise, we built a three-layer artificial neural network, including the input layer, the hidden layer and the output layer with 784, 64, and 10 neurons respectively. The image data source is the standard MNIST handwritten digit recognition data set, each handwritten digit image pixel size is 28 × 28. The same image data was fed into a network without NVS and a network with NVS pre-processing for training (Fig. 5b). The numeral images 7 as an example, noise is added based on the original image, and the noise level ranges from 10% to 80% (Fig. 5c). It can be clearly seen that the image processed by NVS can better reflect the subject in the image. By using the same method to input different digital images into the NVS (Fig. 5d), the NVS based on PRM devices have good pre-noise processing effect on various numbers. Under the condition of 80% noise, the confusion matrix image generated by NVS after 300 training sessions, in which Fig. 5e is the recognition rate of numeral images 7 confusion matrix after 300 training with NVS, and Fig. 5f is the recognition rate of random numeral images confusion matrix after 300 training with NVS. It can be found that the two scenarios of image recognition have better results (More details in the Figs. S20–S28, Supplementary Information). It can be found the image after NVS pre-processing requires less training times, but the recognition effect is better and the recognition rate is higher. This more intuitively illustrates that artificial neural networks containing NVS have more advantages in noise processing (Figs. S29 and S30, Supplementary Information)^36^. Without the participation of NVS, after 300 training sessions, the training sessions and recognition accuracy of images with different noise levels were compared, and it was found that as the noise level increased, the training sessions required for network convergence gradually increased and the recognition accuracy continuously decreased (Fig. 5g). However, after NVS processing, not only the network convergence is faster, but also the recognition rate is more concentrated (Fig. 5h), which is higher than that of images without NVS processing (Fig. 5i). This proves that the NVS system built based on PRM devices can filter the noise in the image well, and then pre-process and recognize the image better, providing a new research idea for the integration of integrated sensors to remove noise.

### Intelligent driving at RPM-NVM mode

**Fig. 6:**
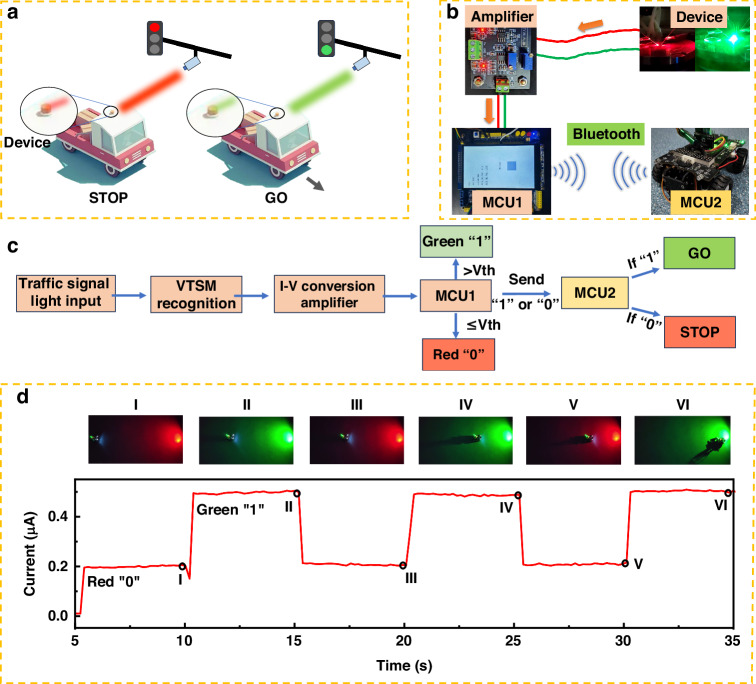
**Intelligent driving system**. **a** Schematic of intelligent recognition of traffic lights**. b** Optical image of traffic lights intelligent recognition circuit system. **c** Flow chart of intelligent recognition system for traffic lights. The response current of the device is input to MCU1 through the IV conversion amplifier for recognition, to determine whether the traffic signal is red or green. Then, it is sent to the intelligent car to complete the “STOP” or “GO” action. **d** Response of PRM device to the traffic light and the motion state of the intelligent car at different time nodes. I to VI indicates the device response and vehicle motion status when the signal light is switched

An intelligent driving system is developed based on the PRM device’s continuous light stability and the fast response of light resolution at different wavelengths. In the future intelligent driving scenario, the traffic light can also be sent through other forms of signals, the intelligent car recognizes the red light in the process of moving, the vehicle stops moving forward - “STOP”, and the car continues to move forward when the signal light turns green - “GO” (Fig. 6a). We use the LTM mode of PRM device to build an intelligent driving system (Fig. 6b), which mainly includes four parts. The first part is the PRM device, which is used as the visual perception part of the car to receive external light signals. The second part is the I-V conversion amplifier module, which converts the photocurrent into a voltage that can be recognized by the microcontroller unit (MCU). The third part is MCU1, which is used to identify the input signal and judge the driving behavior that the signal conforms to. In the fourth part, MCU2, as the control center of the car, receives the signal sent by MCU1 and executes the relevant instructions to complete the relevant actions of intelligent driving. The specific judgment logic is that the PRM device recognizes the traffic light and converts the signal into the corresponding photocurrent value, and the photocurrent value is converted into voltage through the I–V conversion amplifier module. MCU1 reads the voltage and determines whether the signal is green (1) or red (0) according to the threshold voltage set by the program. Send a 1 or 0 to the MCU2 via Bluetooth to control the smart car to move forward or stop (Fig. 6c). Figure 5d shows the motion state of the vehicle after the device receives the signal. The initial state of the intelligent car is the red stop state, from I to II the red light turns green until the green light ends, and the position of the intelligent car changes. II to III, the smart car is in a stopped state, III to IV is in a forward state, IV to V is in a stopped state, and V to VI is in a forward state (Supporting Information Video). The scheme preliminarily proves that PRM devices can be applied to intelligent driving to realize stable and repeatable traffic signal recognition.

## Discussion

Reconfigurable memristors provide a new strategy for dynamic distribution neural networks. We design a simple photonics reconfigurable memristor (PRM) device, and use light programmability to realize the mutual conversion of volatile and non-volatile modes independent of electroforming or limiting current. More importantly, the operating voltage and current in both modes are identically, and the switching voltage can be 100% controllable at any value without limiting current. In addition, the stable performance of the device is demonstrated by the volatile performance sufficient to achieve durability of 10^6^ cycles, and the retention time of 10^4 ^s for 650 nm red light, 520 nm green light and 450 nm blue light in non-volatile mode. We have used the PRM device to simulate the functions of neurons and synapses, and construct applications for a single dynamic distribution neural device at different modes of pulse spiking, short-term memory, and long-term memory. In the neuron mode, the device uses the pulse peak response to realize the recognition of different frequencies and different waveforms of light signals, and finally simulates the important information transmission in navigation - lamp signal, the information is transmitted in the mode of Morse code encryption, and the device completes the real-time and fast decoding. And realized the image recognition based on the characteristic of neuron frequency coding. In the synaptic STM mode, the NVS constructed based on EPSC completed the denoising function in the process of image recognition. When different numbers mixed with 10% to 80% noise, the image was accurately recognized, and the average recognition rate was still as high as 94.31% (the highest 95.33%). The device can also realize LTM mode of synaptic, in which the device can achieve stable state retention. We use this mode to design an intelligent traffic light recognition system, and complete the recognition of the traffic signal in the intelligent car. The PRM device provides a new idea for the realization of dynamic neural networks in the future, and provides a new scheme for the development of highly integrated artificial intelligence.

## Materials and methods

### Fabrication of PRM device

Preparation of ZnO functional layer: The graphene bottom electrode comes from Nanjing MKNANO Tech. Co., LTD., using magnetron sputtering to deposit about 10 nm ZnO on the graphene film, the sputtering steps are: first, the ZnO target and silicon substrate graphene are put into the magnetron sputtering cavity as required; Then open the mechanical pump and molecular pump, pump the vacuum to 2 × 10^-4 ^Pa, turn on the RF source for preheating. Argon: oxygen is 2:1, sputtering power is 10 W, and working pressure is 3 Pa; After the pressure is stable, pre-sputtering is carried out for 5 min to remove surface impurities. After the pre-sputtering is completed, the formal sputtering is carried out for 5 min. The thickness of each layer of the device is shown in TEM Fig. 1b. The Pd top electrode was grown by DC magnetron sputtering, assisted by a mask plate with a diameter of 100 μm, with a power of 10 W, the argon flow rate was 25 sccm, and 3 Pa sputtering pressure for 10 min.

### Device measurement

For electrical measurement, the DC test of the PRM device was performed on an Keysight 2400 semiconductor parameter analyzer. In pulse tests, a Keysight 33,600 A pulse generator served as the power source, and a RIGOL DS6104 oscilloscope was chosen to monitor electrical pulse signals.

### Intelligent driving system

The system mainly consists of two modules, which are built based on STM32, MCU1 is the signal receiving and judging system, and MCU2 is the intelligent vehicle control system, and they communicate with each other through the Bluetooth module. The device connects to the MCU1 system I/O port through the I–V amplifier module, and sets the threshold through the program. When the traffic signal lights up, the device receives and converts the signal, MCU1 determines whether the signal exceeds the threshold, and then sends it to MCU2 through Bluetooth to control the intelligent car, move forward or stop (Fig. 5b–d and Supporting Information Video).

## Supplementary information

Supporting Information for A facile photonics reconfigurable memristor with dynamically allocated neurons and synapses functions

Supporting Vidio

## Data Availability

The data that support the findings of this study are available from the corresponding author upon reasonable request.

## References

[1] Zhang, H. T. et al. Reconfigurable perovskite nickelate electronics for artificial intelligence. *Science* **375**, 533–539 (2022). 35113713 10.1126/science.abj7943

[2] Parisi, G. I. et al. Continual lifelong learning with neural networks: a review. *Neural Netw.* **113**, 54–71 (2019). 30780045 10.1016/j.neunet.2019.01.012

[3] Dang, B. J. et al. Reconfigurable in-sensor processing based on a multi-phototransistor–one-memristor array. *Nat. Electron.* **7**, 991–1003 (2024).

[4] John, R. A. et al. Reconfigurable halide perovskite nanocrystal memristors for neuromorphic computing. *Nat. Commun.* **13**, 2074 (2022). 35440122 10.1038/s41467-022-29727-1 PMC9018677

[5] Zhong, Y. N. et al. Dynamic memristor-based reservoir computing for high-efficiency temporal signal processing. *Nat. Commun.* **12**, 408 (2021). 33462233 10.1038/s41467-020-20692-1 PMC7814066

[6] Aguirre, F. et al. Hardware implementation of memristor-based artificial neural networks. *Nat. Commun.* **15**, 1974 (2024). 38438350 10.1038/s41467-024-45670-9 PMC10912231

[7] Zhao, J. H. et al. Neural morphology perception system based on antiferroelectric AgNbO_3_ neurons. *InfoMat* **7**, e12637 (2025).

[8] Yan, X. B. et al. A multimode‐fused sensory memory system based on a robust self‐assembly nanoscaffolded BaTiO_3_: Eu_2_O_3_ memristor. *InfoMat* **5**, e12429 (2023).

[9] Wang, T. Y. et al. Reconfigurable neuromorphic memristor network for ultralow-power smart textile electronics. *Nat. Commun.* **13**, 7432 (2022). 36460675 10.1038/s41467-022-35160-1 PMC9718838

[10] Guo, T. et al. Versatile memristor for memory and neuromorphic computing. *Nanoscale Horiz.* **7**, 299–310 (2022). 35064257 10.1039/d1nh00481f

[11] He, N. et al. Multifunctional Ag–In–Zn–S/Cs_3_Cu_2_Cl_5_‐based memristors with coexistence of non‐volatile memory and volatile threshold switching behaviors for neuroinspired computing. *Adv. Electron. Mater.* **9**, 2201038 (2023).

[12] Wang, T. Y. et al. Reconfigurable optoelectronic memristor for in-sensor computing applications. *Nano Energy* **89**, 106291 (2021).

[13] Shan, X. Y. et al. Plasmonic optoelectronic memristor enabling fully light‐modulated synaptic plasticity for neuromorphic vision. *Adv. Sci.* **9**, 2104632 (2022). 10.1002/advs.202104632 PMC8867191 34967152

[14] Zha, J. et al. Electronic/optoelectronic memory device enabled by tellurium‐based 2D van der waals heterostructure for in‐sensor reservoir computing at the optical communication band. *Adv. Mater.* **35**, 2211598 (2023). 10.1002/adma.202211598 36857506

[15] Lee, J. et al. Light-enhanced molecular polarity enabling multispectral color-cognitive memristor for neuromorphic visual system. *Nat. Commun.* **14**, 5775 (2023). 37723149 10.1038/s41467-023-41419-y PMC10507016

[16] Zhang, J. Y. et al. Recent progress in photonic synapses for neuromorphic systems. *Adv. Intell. Syst.* **2**, 1900136 (2020).

[17] Zhou, F. C. et al. Optoelectronic resistive random access memory for neuromorphic vision sensors. *Nat. Nanotechnol.* **14**, 776–782 (2019). 31308498 10.1038/s41565-019-0501-3

[18] Feng, X. W., Liu, X. K. & Ang, K. W. 2D photonic memristor beyond graphene: progress and prospects. *Nanophotonics* **9**, 1579–1599 (2020).

[19] Wang, Y. et al. Photonic synapses based on inorganic perovskite quantum dots for neuromorphic computing. *Adv. Mater.* **30**, e1802883 (2018). 30063261 10.1002/adma.201802883

[20] Guo, Y. B. & Zhu, L. Q. Recent progress in optoelectronic neuromorphic devices. *Chin. Phys. B* **29**, 078502 (2020).

[21] Ilyas, N. et al. Nanostructured materials and architectures for advanced optoelectronic synaptic devices. *Adv. Funct. Mater.* **32**, 2110976 (2022).

[22] Yuan, R. et al. A calibratable sensory neuron based on epitaxial VO_2_ for spike-based neuromorphic multisensory system. *Nat. Commun.* **13**, 3973 (2022). 35803938 10.1038/s41467-022-31747-w PMC9270461

[23] Yuan, R. et al. A neuromorphic physiological signal processing system based on VO_2_ memristor for next-generation human-machine interface. *Nat. Commun.* **14**, 3695 (2023). 37344448 10.1038/s41467-023-39430-4 PMC10284901

[24] Choi, Y. et al. Physically defined long-term and short-term synapses for the development of reconfigurable analog-type operators capable of performing health care tasks. *Sci. Adv.* **9**, eadg5946 (2023). 37406117 10.1126/sciadv.adg5946 PMC10321737

[25] Zhang, Y. et al. Evolution of the conductive filament system in HfO_2_-based memristors observed by direct atomic-scale imaging. *Nat. Commun.* **12**, 7232 (2021). 34903752 10.1038/s41467-021-27575-z PMC8668918

[26] Dang, V. Q. et al. Ultrahigh responsivity in graphene-zno nanorod hybrid uv photodetector. *Small* **11**, 3054–3065 (2015). 25703808 10.1002/smll.201403625

[27] Liu, S. et al. Strain modulation in graphene/zno nanorod film schottky junction for enhanced photosensing performance. *Adv. Funct. Mater.* **26**, 1347–1353 (2016).

[28] Yang, T. T. et al. Green energy and content-aware data transmissions in maritime wireless communication networks. *IEEE Trans. Intell. Transport. Syst.* **16**, 751–762 (2015).

[29] Alqurashi, F. S. et al. Maritime communications: a survey on enabling technologies, opportunities, and challenges. *IEEE Internet Things J.* **10**, 3525–3547 (2023).

[30] Song, L. K. et al. Spiking neurons with neural dynamics implemented using stochastic memristors. *Adv. Electron. Mater.* **10**, 2300564 (2024).

[31] Zhu, C. G. et al. Optical synaptic devices with ultra-low power consumption for neuromorphic computing. *Light Sci. Appl.* **11**, 337 (2022). 36443284 10.1038/s41377-022-01031-z PMC9705294

[32] Li, Y. et al. Flexible artificial optoelectronic synapse based on lead‐free metal halide nanocrystals for neuromorphic computing and color recognition. *Adv. Sci.* **9**, 2202123 (2022). 10.1002/advs.202202123 PMC9353487 35661449

[33] Schreiber, W. F. *Fundamentals of Electronic Imaging Systems: Some Aspects of Image Processing* 3rd edn (Springer, 1999).

[34] Lukas, J., Fridrich, J. & Goljan, M. Digital camera identification from sensor pattern noise. *IEEE Trans. Inf. Forensics Security* **1**, 205–214 (2006).

[35] Chen, Y. L. et al. Image processing for denoising using composite adaptive filtering methods based on rmse. *Open J. Appl. Sci.* **14**, 660–675 (2024).

[36] Chen, J. W. et al. Optoelectronic graded neurons for bioinspired in-sensor motion perception. *Nat. Nanotechnol.* **18**, 882–888 (2023). 37081081 10.1038/s41565-023-01379-2

