# Supplementary material for: A facile photonics reconfigurable memristor with dynamically allocated neurons and synapses functions
Source: Light Sci Appl. 2025 Aug 12;14:269. doi: 10.1038/s41377-025-01928-5 (PMC12340011; doi:10.1038/s41377-025-01928-5)
Supplement: Supplementary file 1 — Supporting Information for A facile photonics reconfigurable memristor with dynamically allocated neurons and synapses functions [file 41377_2025_1928_MOESM1_ESM.docx]

**Supplementary Information**

**A facile photonics reconfigurable memristor with dynamically allocated neurons and synapses functions**

Zhenyu Zhou ^1#^, Lulu Wang ^1#^, Gongjie Liu ^1, 2#^, Yuchen Li ^1^, Zhiyuan Guan ^1^, Zixuan Zhang ^1^, Pengfei Li ^1^, Yifei Pei ^1^, Jianhui Zhao ^1^, Jiameng Sun ^1^, Yahong Wang ^1^, Yiduo Shao ^1^ and Xiaobing Yan *^, 1^

^1^School of Life Sciences, Institute of Life Science and Green Development, Key Laboratory of Brain-Like Neuromorphic Devices and Systems of Hebei Province, College of Electron and Information Engineering, Hebei University, Baoding, the People's Republic of China.

^2^School of Electronic Science and Engineering, Xiamen University, Xiamen, 361005, China.

^#^ These authors contributed equally to the work.

**Correspondence**

Xiaobing Yan, School of Life Sciences, Institute of Life Science and Green Development, Key Laboratory of Brain-Like Neuromorphic Devices and Systems of Hebei Province, College of Electron and Information Engineering, Hebei University, Baoding 071002, the People's Republic of China. Email: yanxiaobing@ime.ac.cn


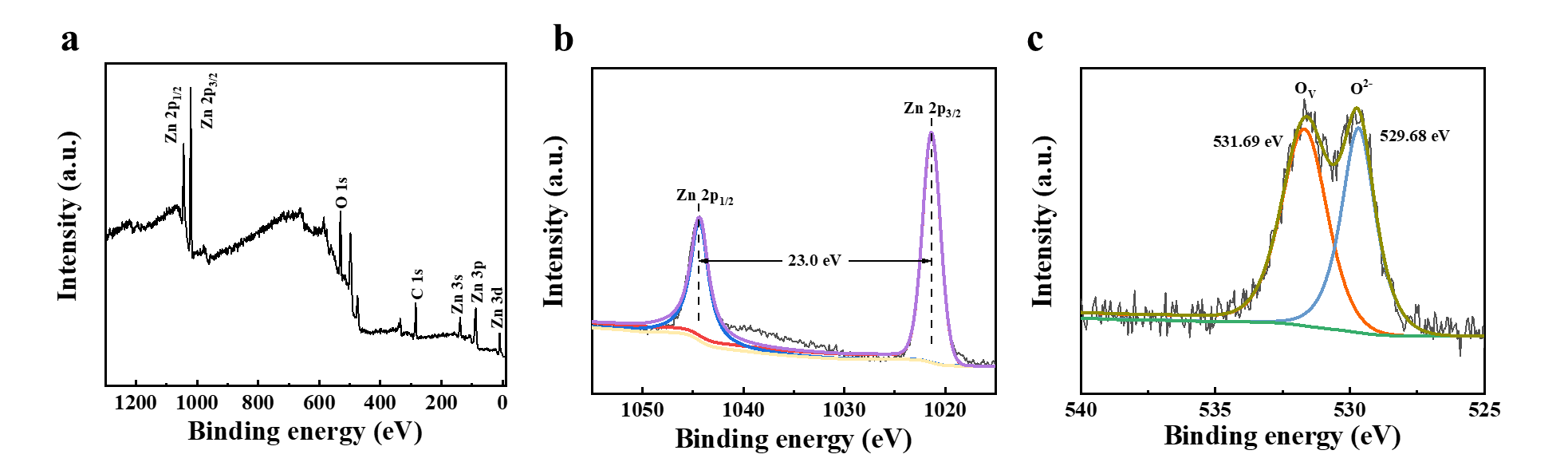
 **Fig. S1** **a** XPS full spectrum of ZnO film; **b** XPS spectrum of 2p nuclear level of Zn element; **c** The 1s nuclear level XPS spectrum of the O element.

The chemical properties and binding energies of ZnO films were characterized by XPS, and the binding energies of all identified peaks were calibrated by C1s energy at 284.8eV. Elements including Zn 2p and O 1s are detected by XPS, as shown in Fig.S1a. Fig. S1b shows the Zn 2p spectrum. The two peaks in the figure correspond to Zn 2p_1/2_ and Zn 2p_3/2_ respectively, and their binding energies are 1044.1 eV and 1021.1 eV, respectively. The energy difference between the two peaks is 23 eV, which is consistent with the typical binding energy of Zn^2+^ in ZnO, confirming that the valence state of Zn is +2 and exists in the form of ZnO. The spectral diagram of O 1s (Fig. S1c) shows that the binding energy peaks of O 1s are asymmetrical, with two peaks at 531.69 eV and 529.68 eV. Among them, the peaks at 529.68 eV and 531.69 eV can be attributed to lattice oxygen (OL) and oxygen vacancy (OV), respectively, which is consistent with the results obtained in the literature^1^. The presence of oxygen vacancy can provide a new defect level for ZnO films, which is conducive to their response in the visible light range^2,3^.


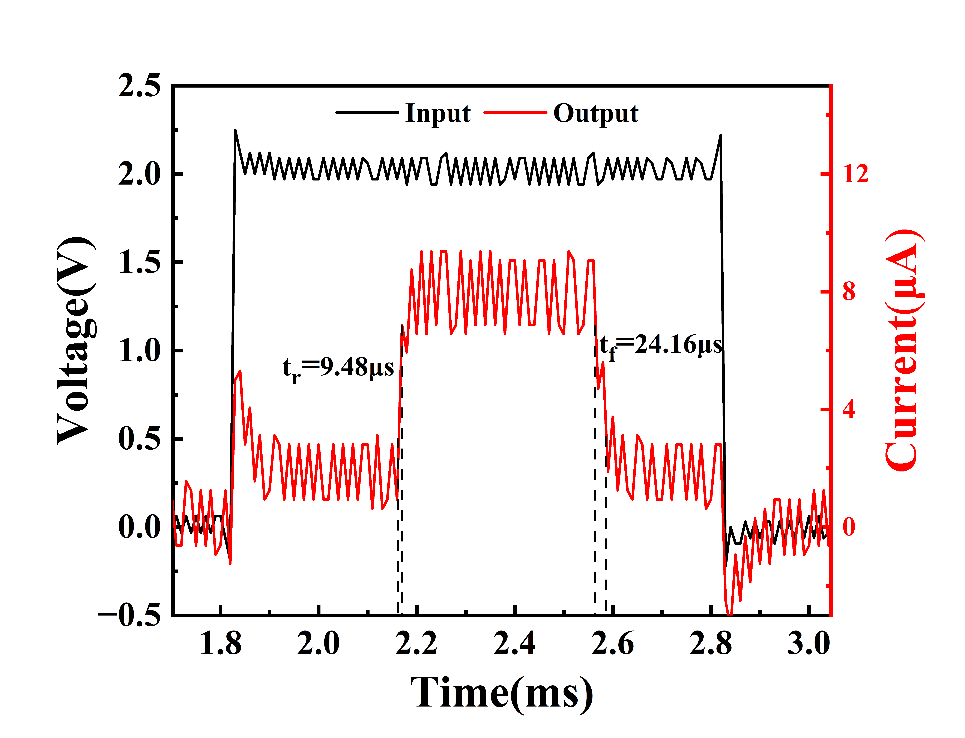


**Fig. S2.** Switching speed of the PRM device under the combined action of electrical pulse and light pulse. the electrical pulse width is 1 ms, the amplitude is 2V, and the width of the optical pulse is 500μs, and the final measured opening speed is 9.48μs, and the closing speed is 24.16μs.


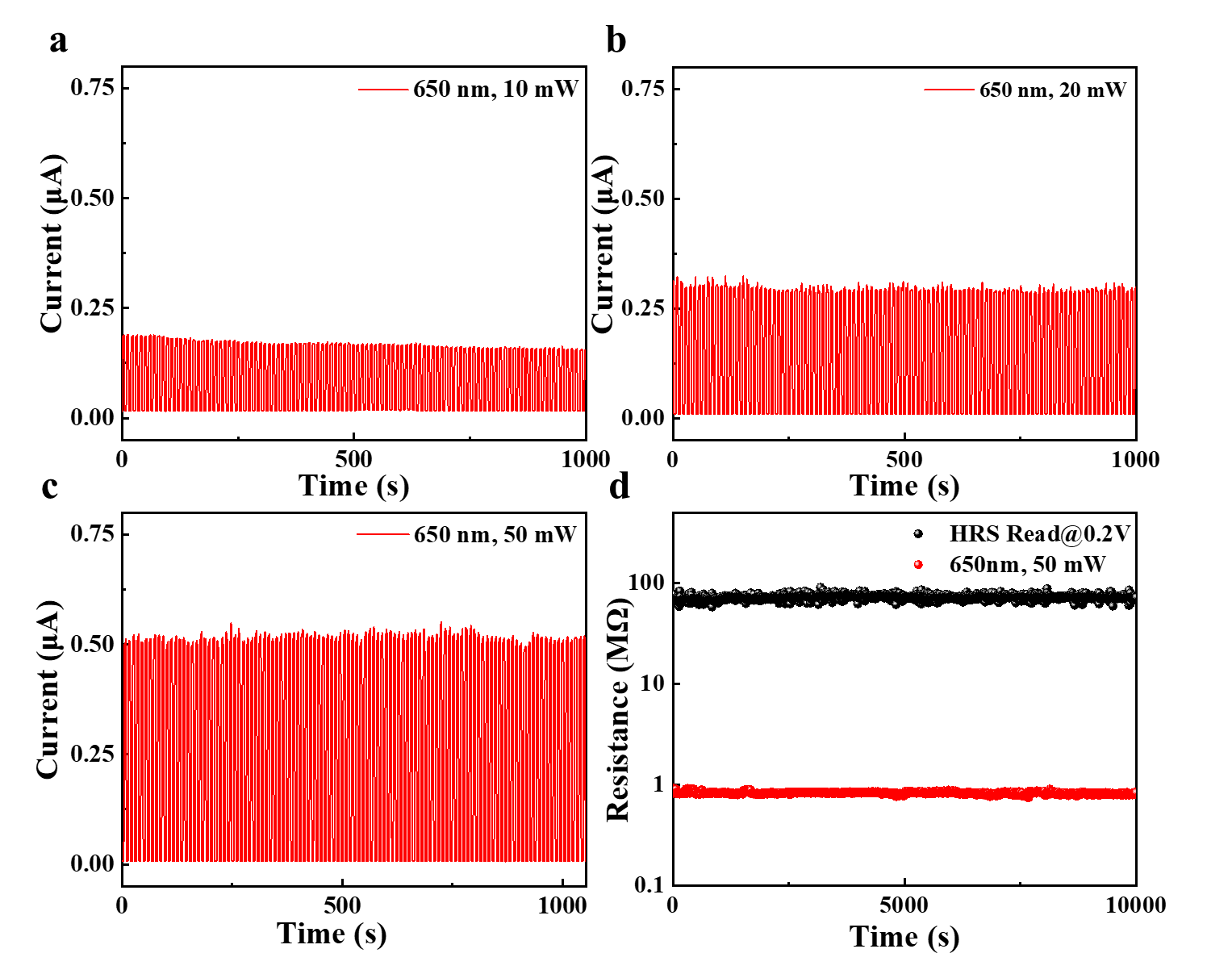


**Fig. S3. a-c** The I-T curves are obtained under light pulse (width 5s, interval 5 s) period under light irradiation with wavelength of 650 nm and power of 10 mW, 20 mW and 50 mW, respectively. **d** The retention characteristics of the device under light irradiation with a wavelength of 650 nm and a power of 50 mW.


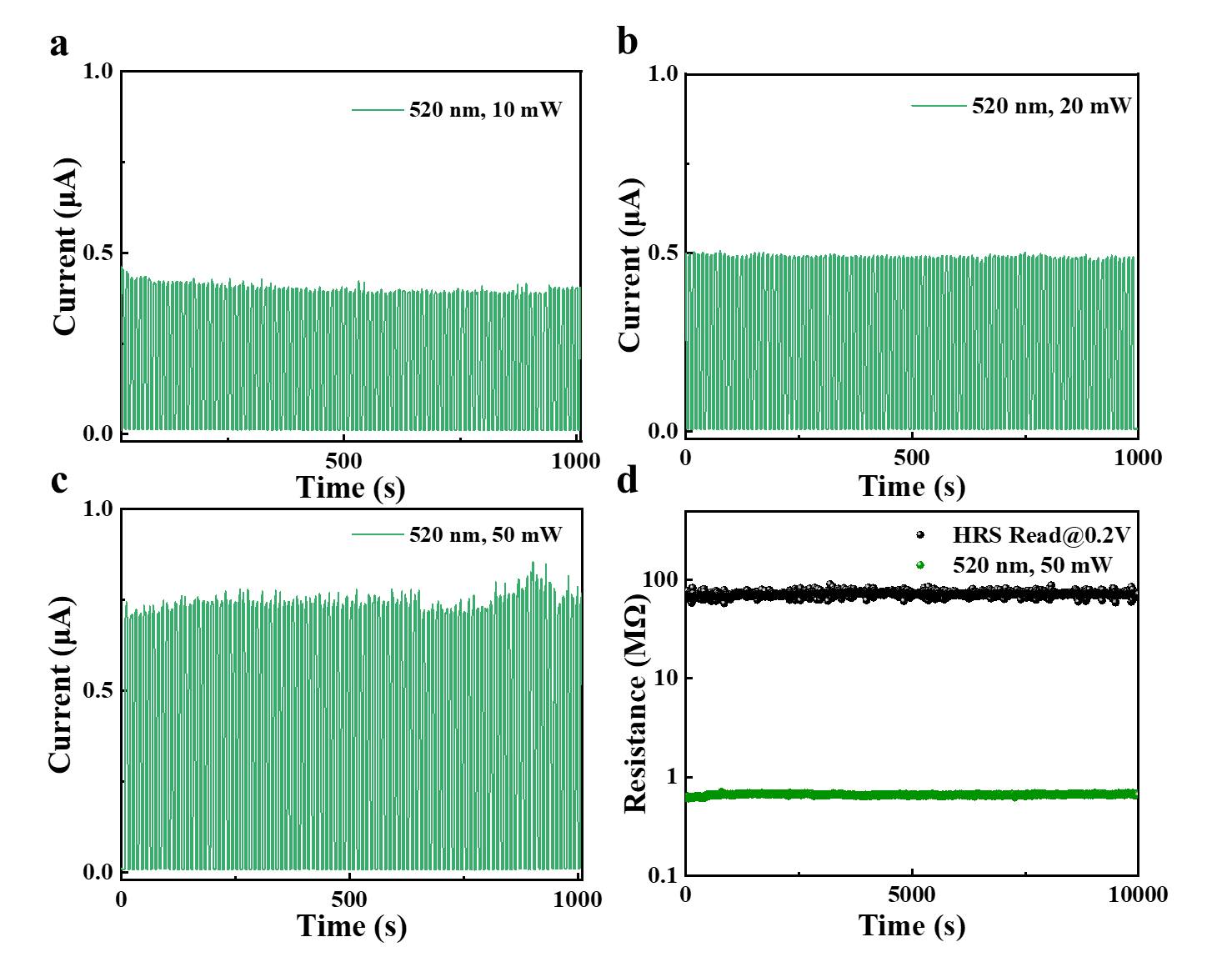


**Fig. S4. a-c** The I-T curves are obtained under light pulse (width 5s, interval 5 s) period under light irradiation with wavelength of 520 nm and power of 10 mW, 20 mW and 50 mW, respectively. d The retention characteristics of the device under light irradiation with a wavelength of 520 nm and a power of 50 mW.


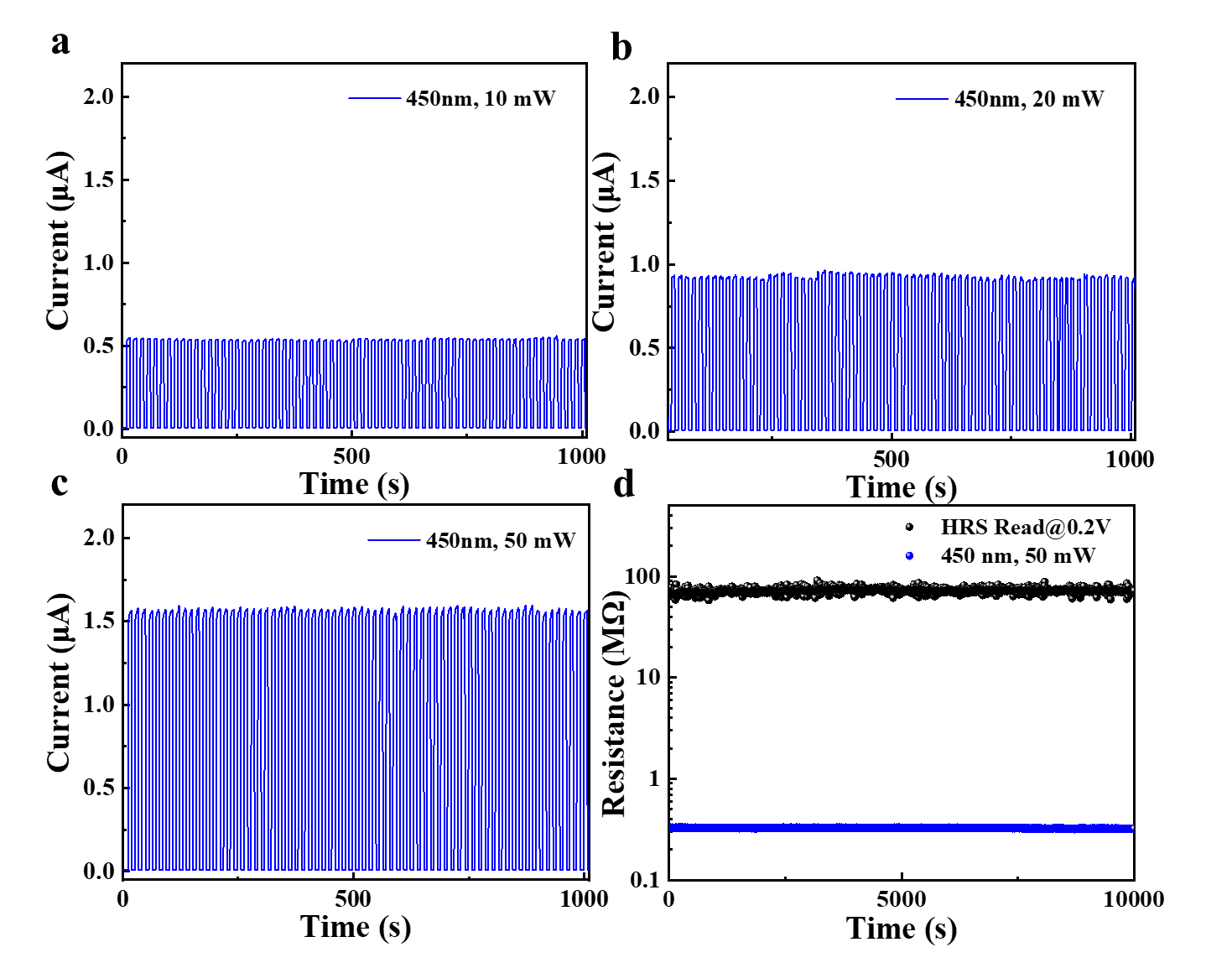


**Fig. S5.** **a-c** The I-T curves are obtained under light pulse (width 5s, interval 5 s) period under light irradiation with wavelength of 450 nm and power of 10 mW, 20 mW and 50 mW, respectively. **d** The retention characteristics of the device under light irradiation with a wavelength of 450 nm and a power of 50 mW.


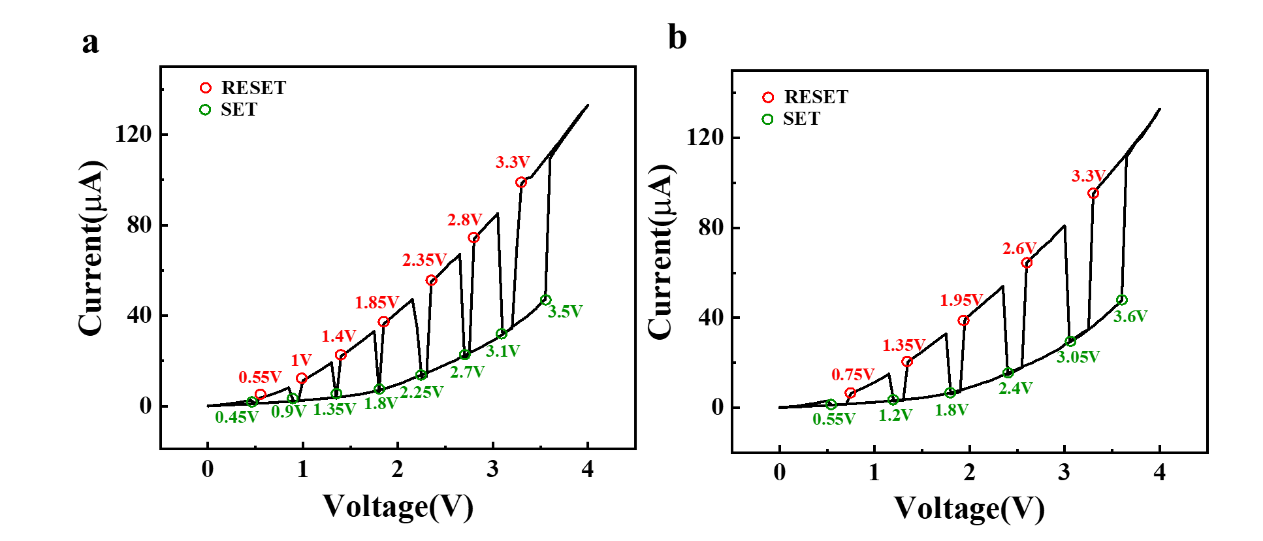


**Fig. S6.** **a and b** Conversion of high and low resistance states at any voltage position in DC scanning mode. It further proves that the PRM device can be turned on and off at any position by controlling light, and does not require any limiting current for regulation, and more importantly, it does not require electroforming operations to intervene in advance in the formation of conductive channels inside the film. This will provide convenience for the application of the device in the hardware circuit and reduce the cost.


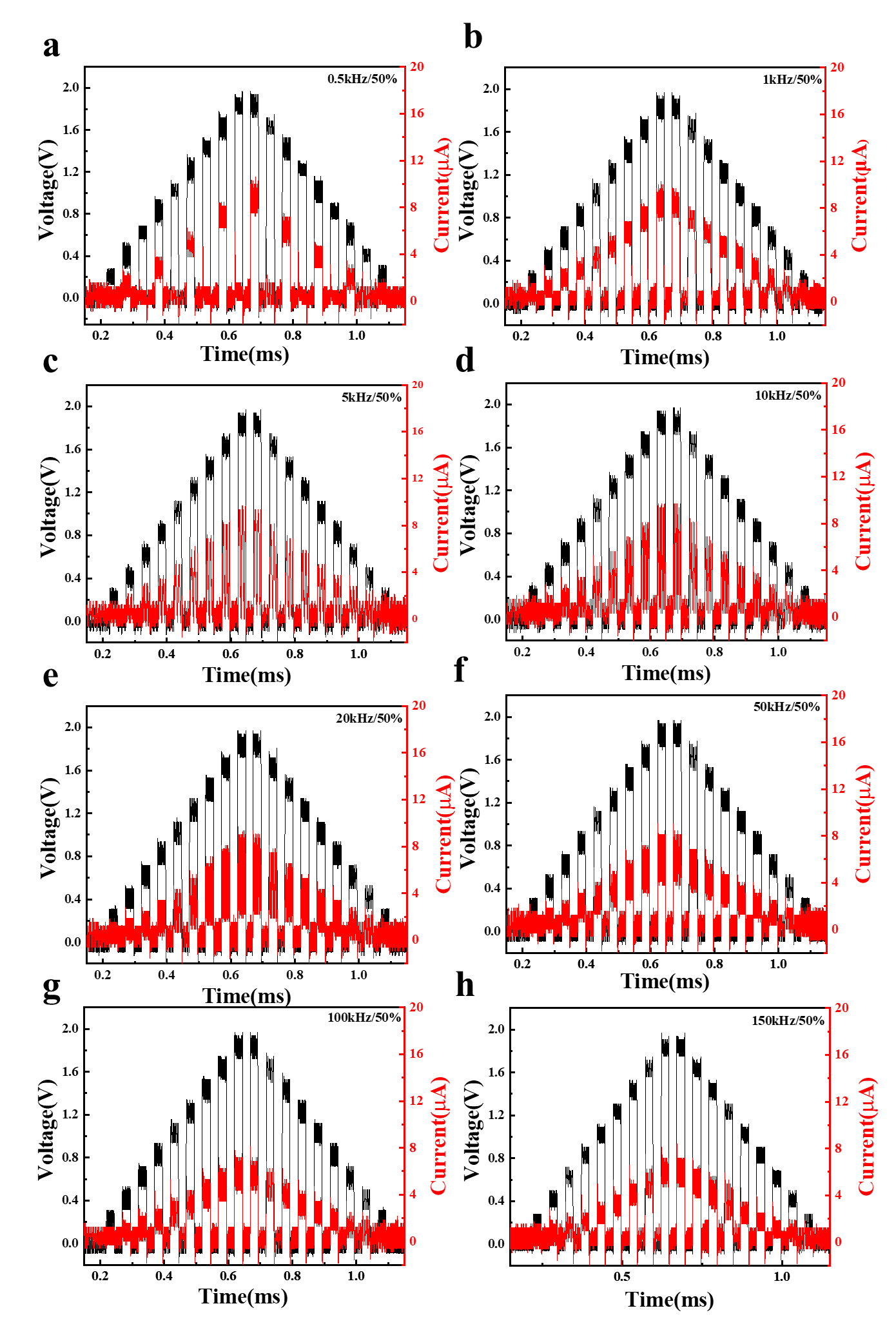


**Fig. S7.** The switching state of the device at any voltage and frequency in pulse mode. The electrical pulse from 0.2V to 1.8V has a step length of 0.2V, light pulse is 0.5kHz to150kHz with 50% duty. It is proved that the device can control the threshold phenomenon in different pulse scenarios。


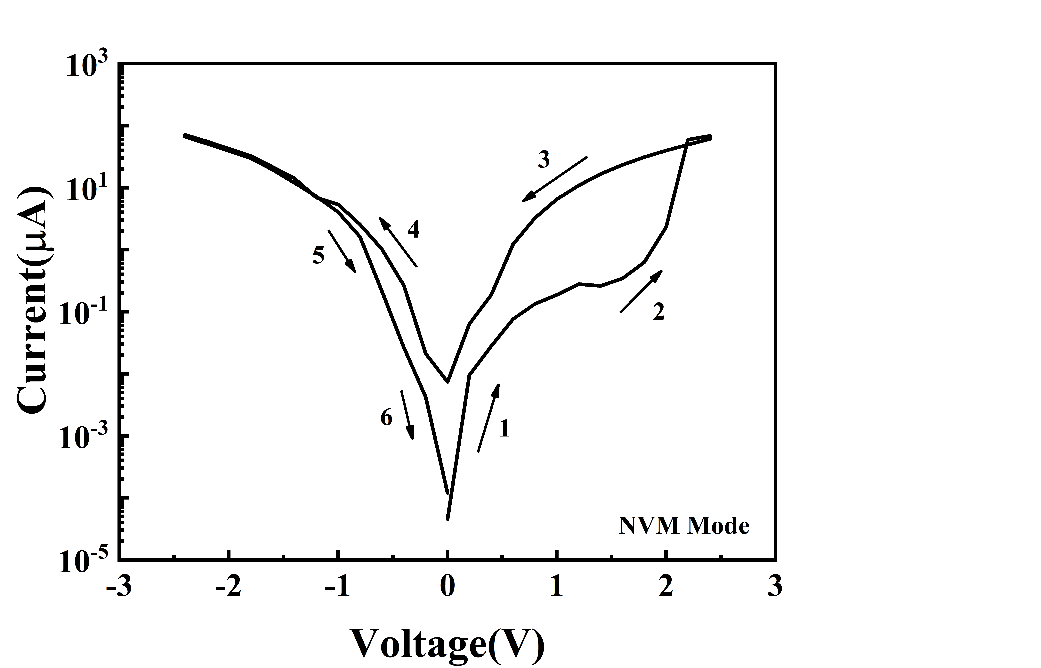


**Fig. S8**. The complete I-V log curve under DC test clearly accords with the basic electrical characteristics of the memristor, that is, the current trend is 1to 6, which means that the device has non-volatile characteristics.


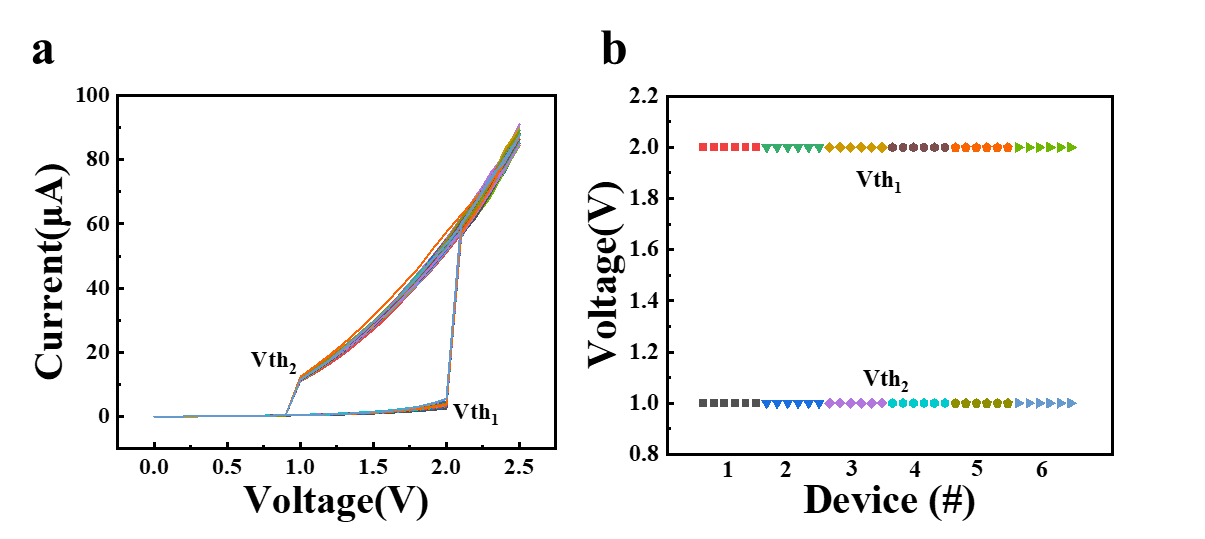


**Fig. S9.** **a** The device-to-device performance for the threshold switching behaviors. The I-V curves of six different devices were tested six times; **b** Six different threshold devices on voltage (Vth1) and off voltage (Vth2) statistics.


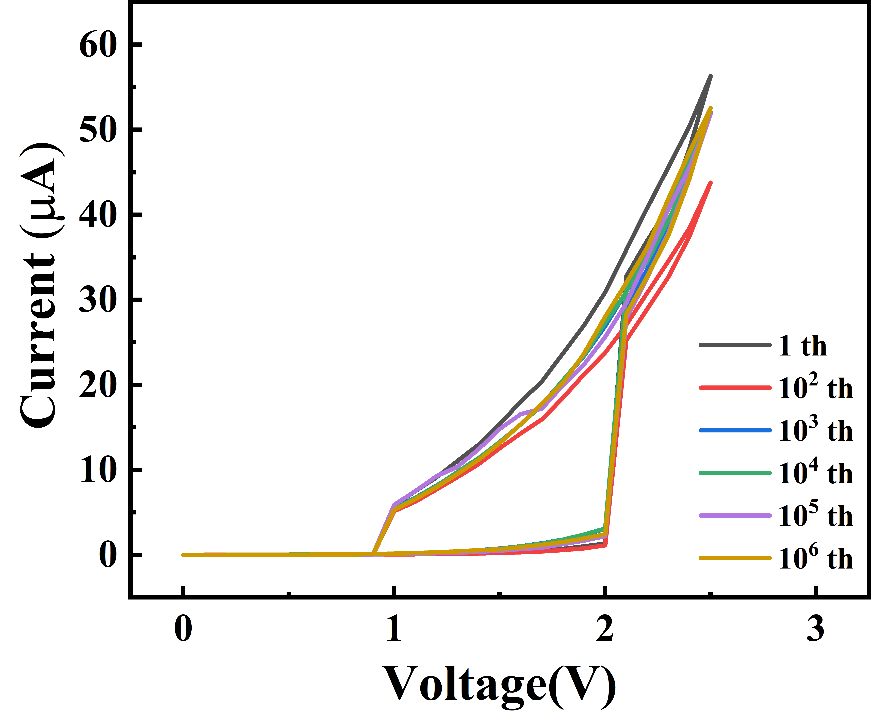


**Fig. S10.** Durability of devices in photoelectric mode. The I-V curves after a maximum of 10^6^ times photoelectric switches can still achieve a stable resistance transition under the Vth_1_and Vth_2_ voltage.


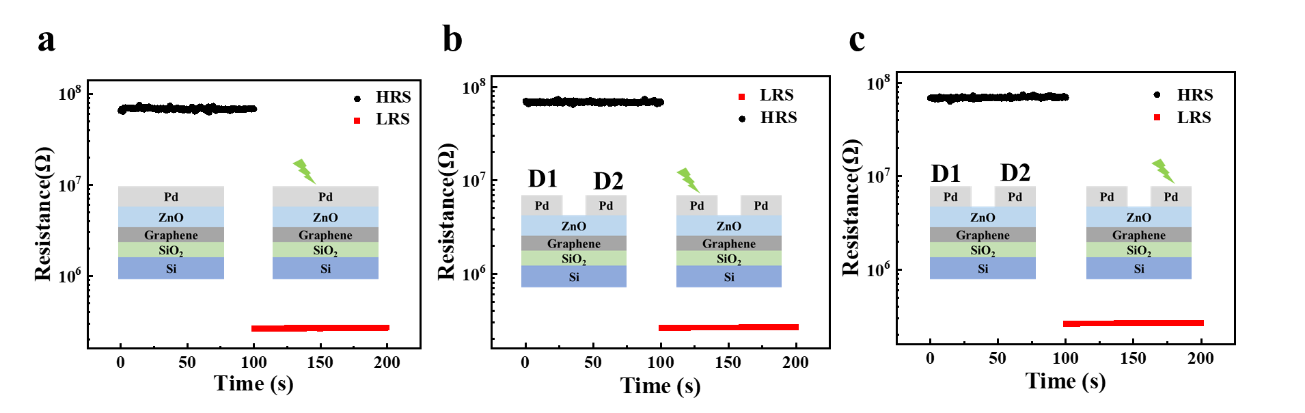


**Fig. S11** Different regional resistance states and light dependent behaviors. **a** HRS and LRS of intact electrode devices. **b** HRS and LRS of the device 1(D1), after the electrode is cut. **c** HRS and LRS of the device 2 (D2), after the electrode is cut. These behaviors prove that the HRS and LRS of the device does not depend on the conductive filament.


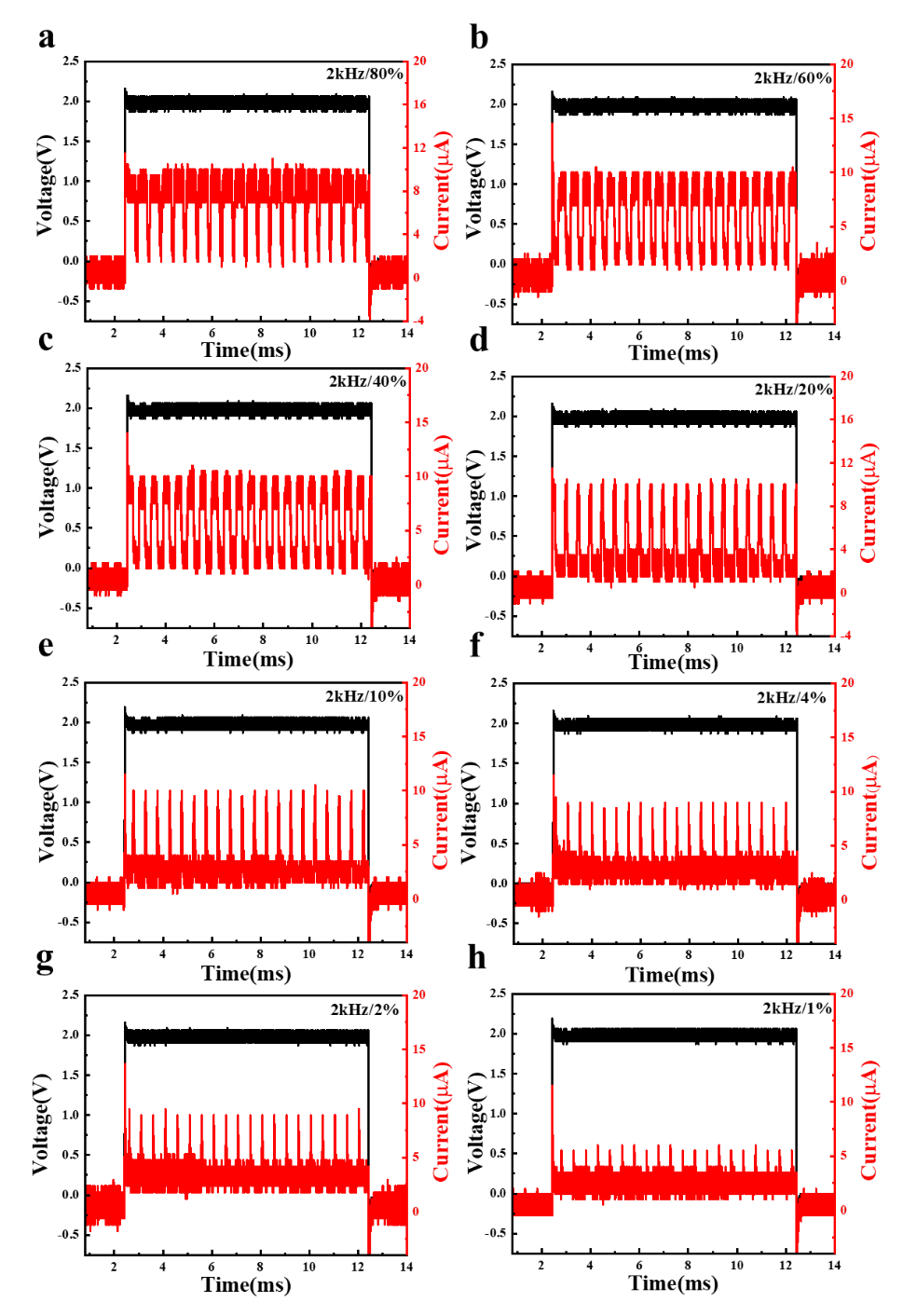


**Fig. S12.** The device applied the light pulse with different duty cycle frequency of 2kHz, the neuron fire characteristics were realized, and the larger the duty cycle, the longer the fire time. This paper makes full use of this feature to simulate the dots and bars of Morse code.


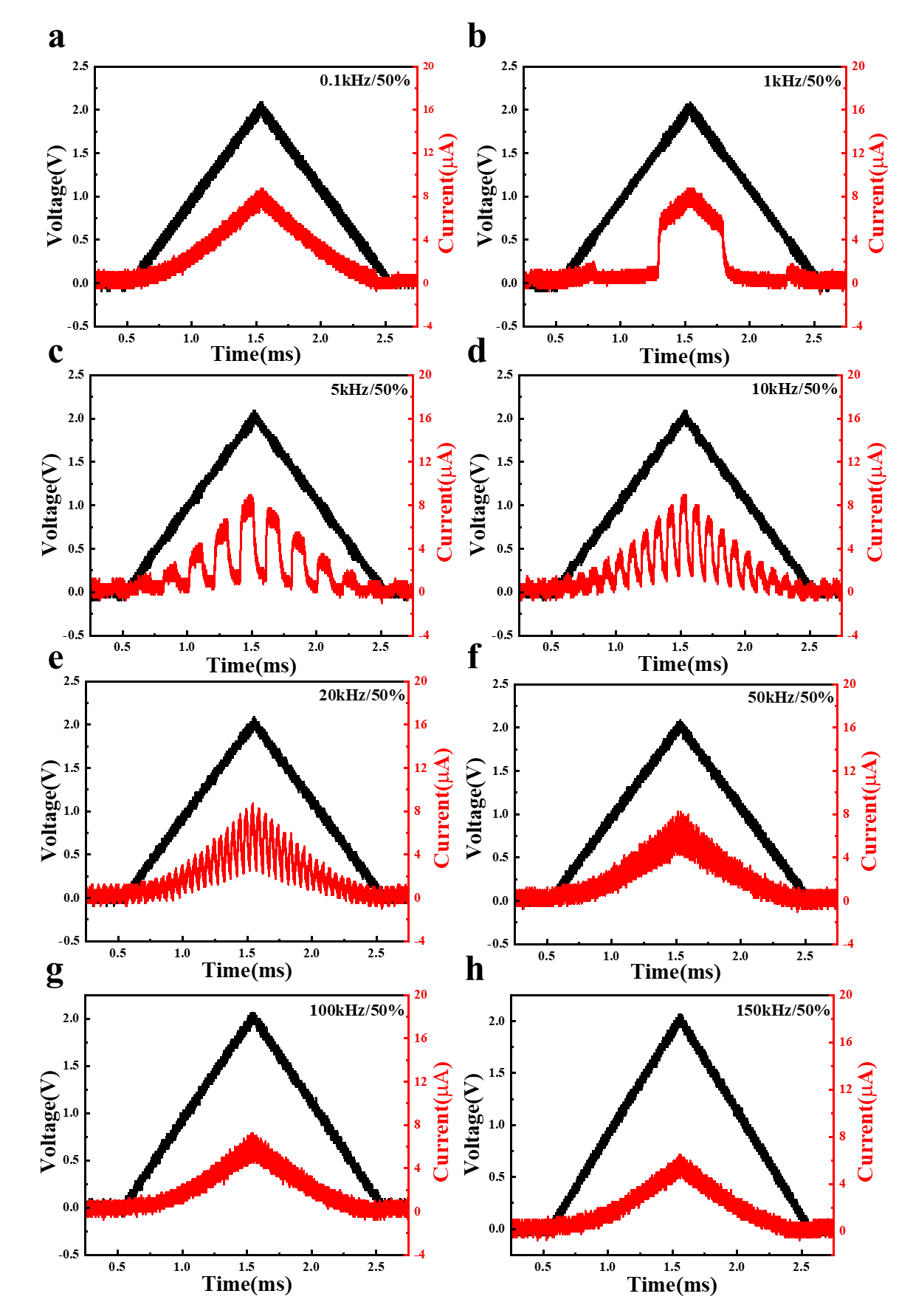


**Fig. S13.** The relationship between triangular wave and light pulse frequency.


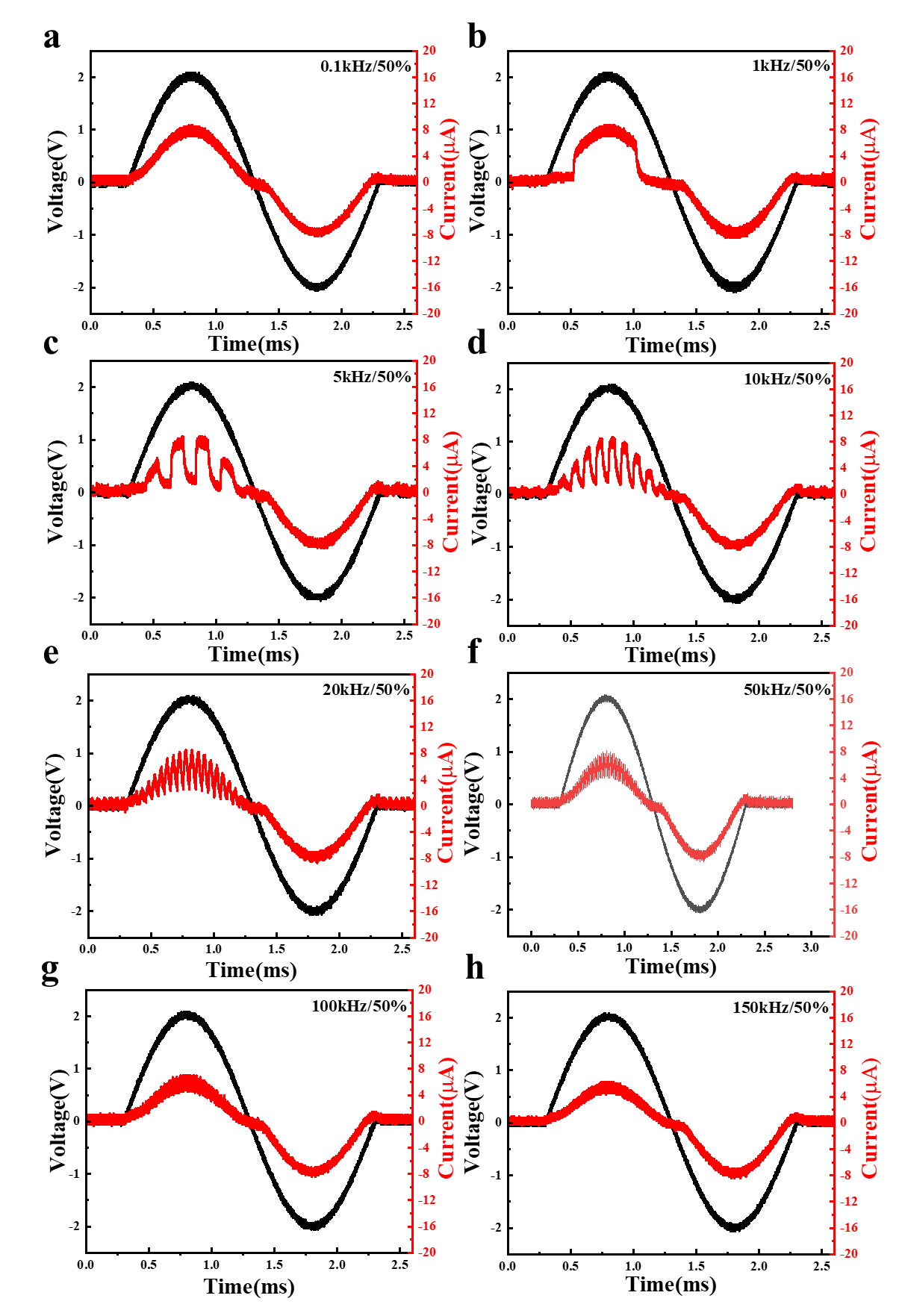


**Fig. S14.** The relationship between sine wave and light pulse frequency.


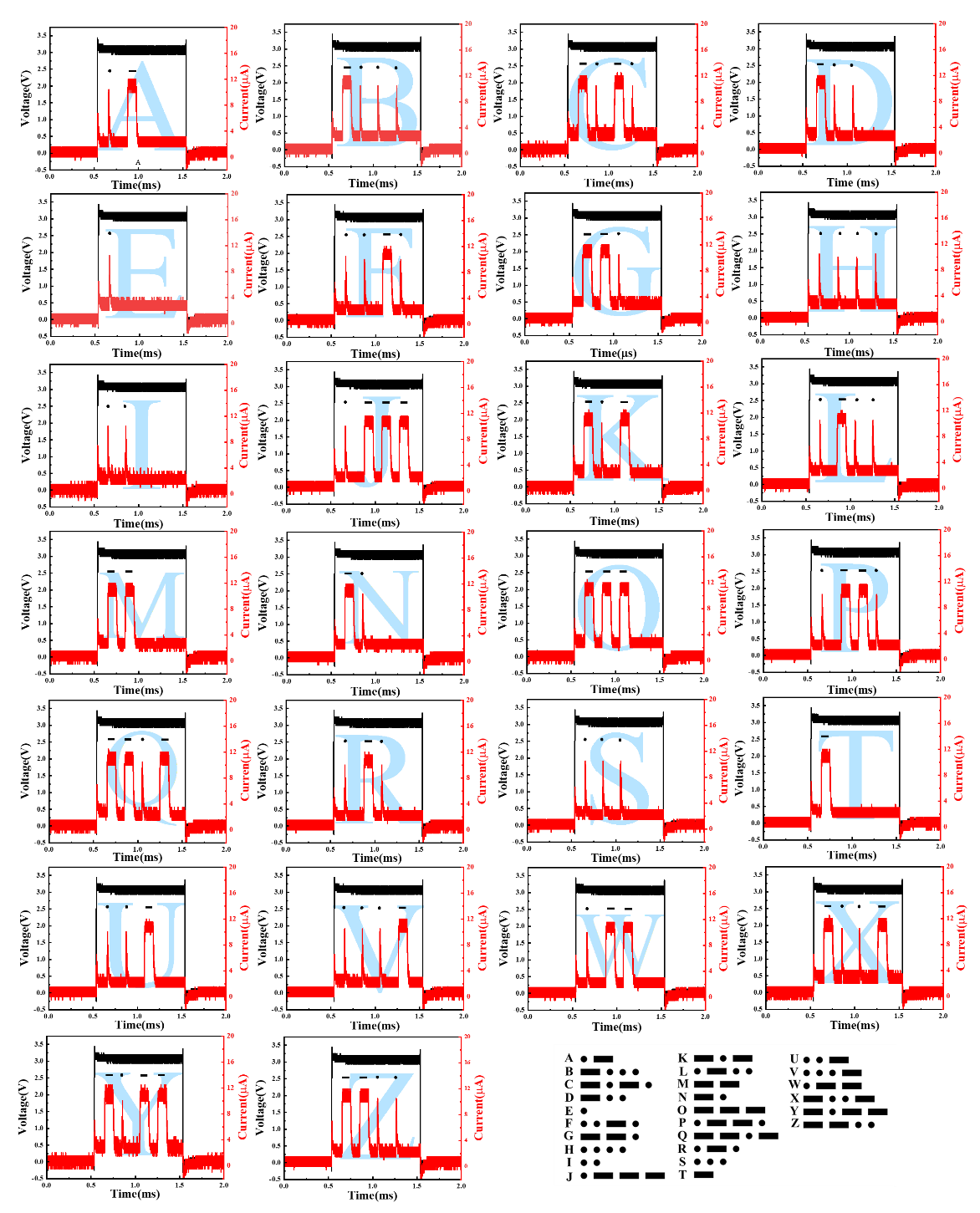


**Fig. S15.** In PRM-VTSM mode, the letters A to Z are encoded to drive the corresponding Morse coded pulse of light emission respectively, and the waveform diagram is obtained through the oscilloscope. 2% of the 2kHz light is used to simulate the Morse coded short point signal “.”, and 4% of the 2kHz light is used to simulate the long signal “—”.


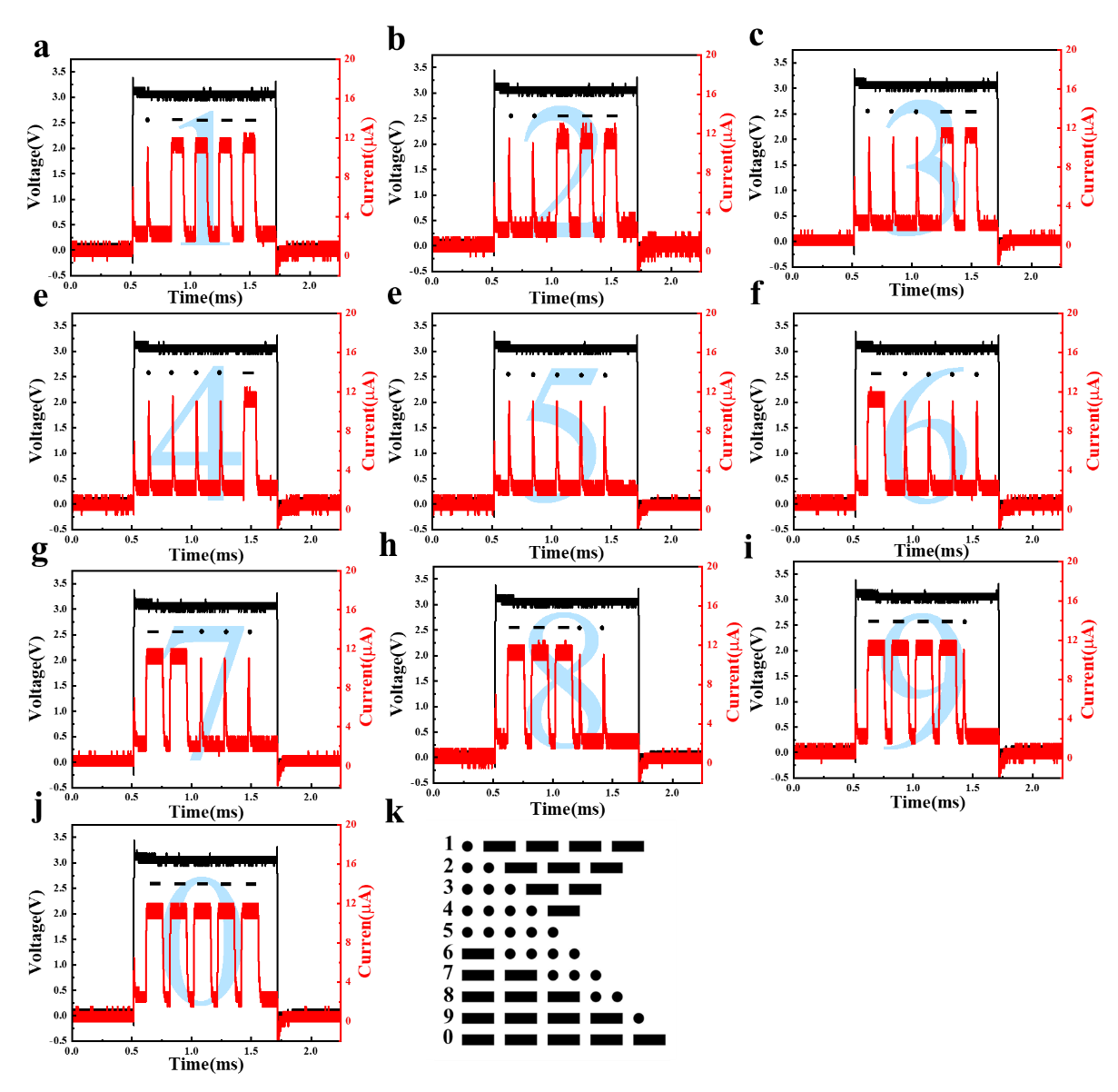


**Fig. S16. a to j** In PRM-VTSM mode, the numbers 0 to 9 are encoded to drive the corresponding Morse coded pulse of light emission respectively, and the waveform diagram is obtained through the oscilloscope. **k** Each number corresponds to a Morse code.


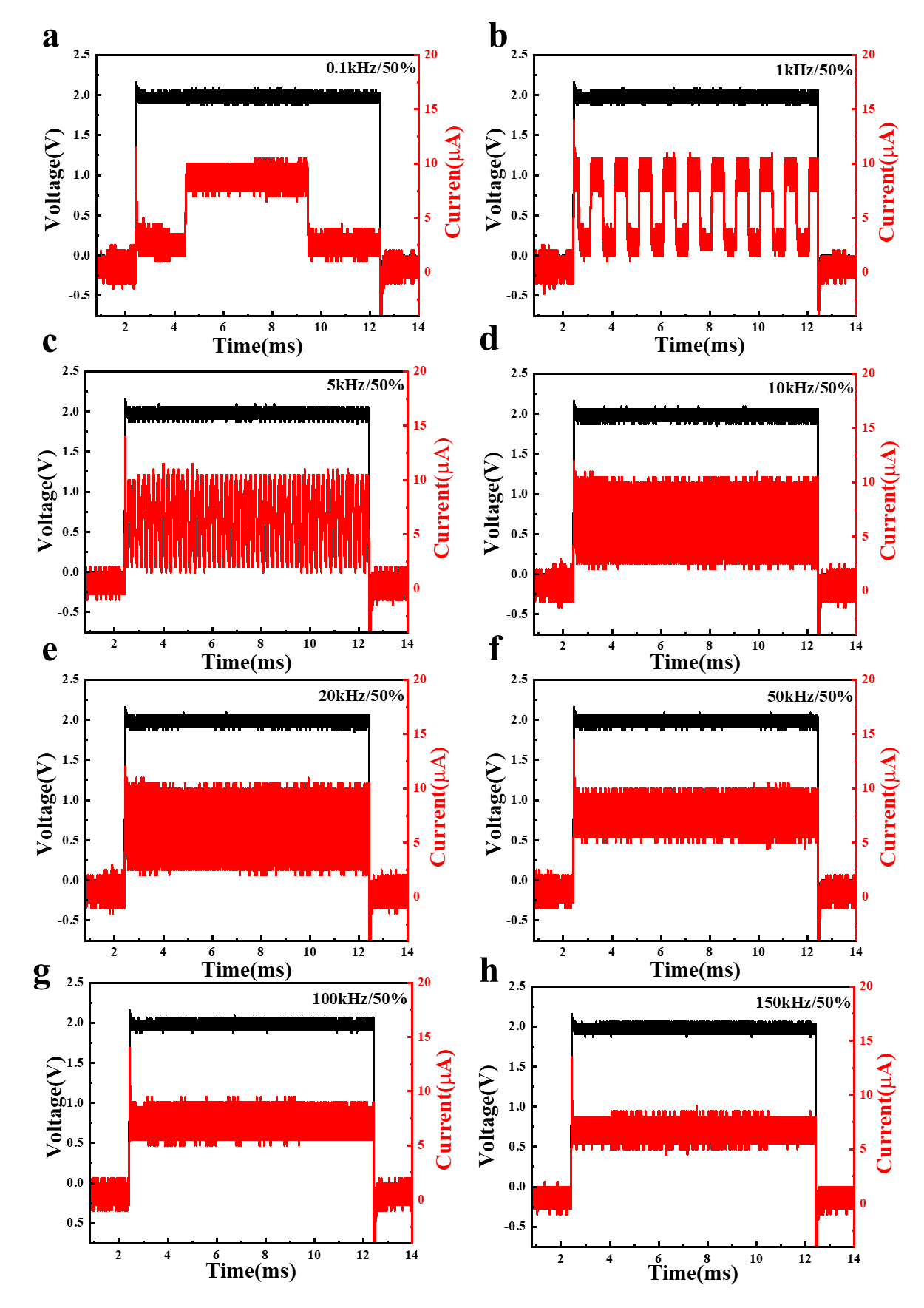


**Fig. S17.** The relationship between square wave and light pulse frequency. Under a voltage pulse (width 10ms, amplitude 2v), changing the frequency of the light revealed that as the frequency increased, the switching times of the device also increased, and the current in the device gradually shifted towards the current of lower resistance value.


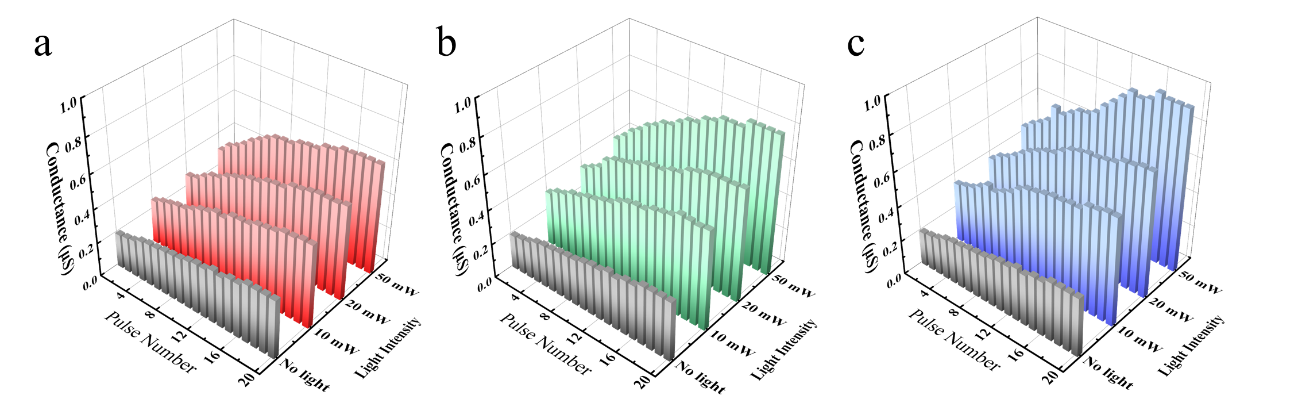


**Fig. S18.** Effect of applied light on the control of device conductance. **a** Applying optical stimulation with wavelength of 650 nm and different power (10, 20, 50mW) to regulate the conductance of the device; **b** Applying the wavelength of 520 nm, different power (10, 20, 50mW) of light stimulation to the device conductance regulation; **c** Applying optical stimulation with wavelength of 450 nm and different power (10, 20, 50mW) to regulate the conductance of the device. In PRM-NVM mode, it is necessary to keep the light normally open and then apply 20 electrical pulses (4V, 500ns, 500ns) to regulate the device conductance. It is found that the conductance increases gradually with the increase of the number of pulses. This is also very much in line with synaptic STM.


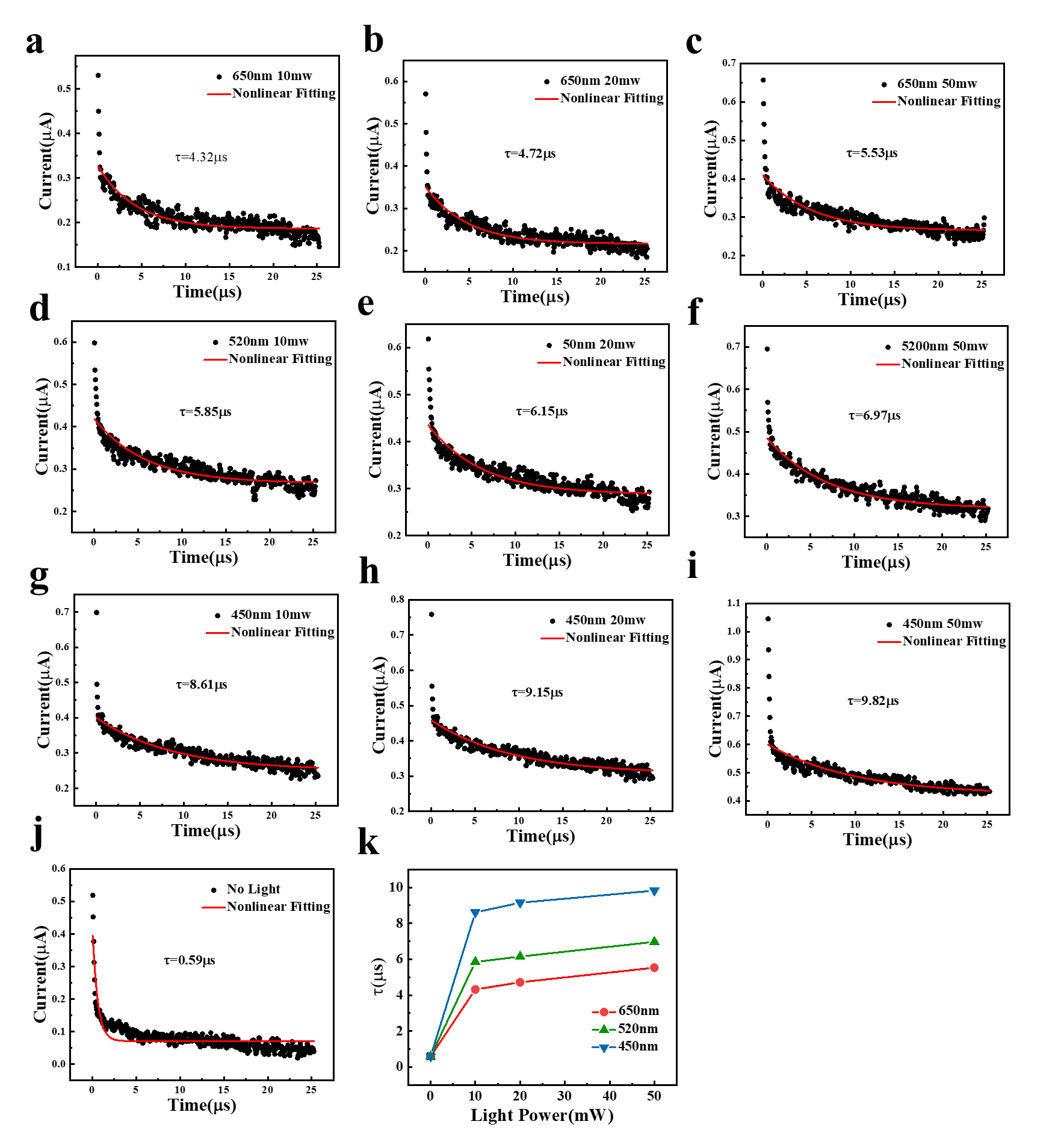


**Fig. S19. a-i** The EPSC generated under the combined action of light and electric pulses of different wavelengths and energies was fitted, wherein relaxation time t represented the forgetting ability of synapses, and the greater relaxation time represented the longer synaptic memory. **j** Relaxation time fitting of device EPSC in the absence of light**.**  **k** The relationship between relaxation time and different intensities of light.

The relationship between forgetting behavior of memristor devices and STM of synapses is used to describe with exponential decay equation as:

M(t) = M_e_ + (M_0_ − M_e_) exp(− t/τ ) (1)

where M_0_ is the initial memory state, M_e_ is the steady state of memory, and τ is the time constant of relaxation process. If the τ value is larger, the forgetting will become slower.


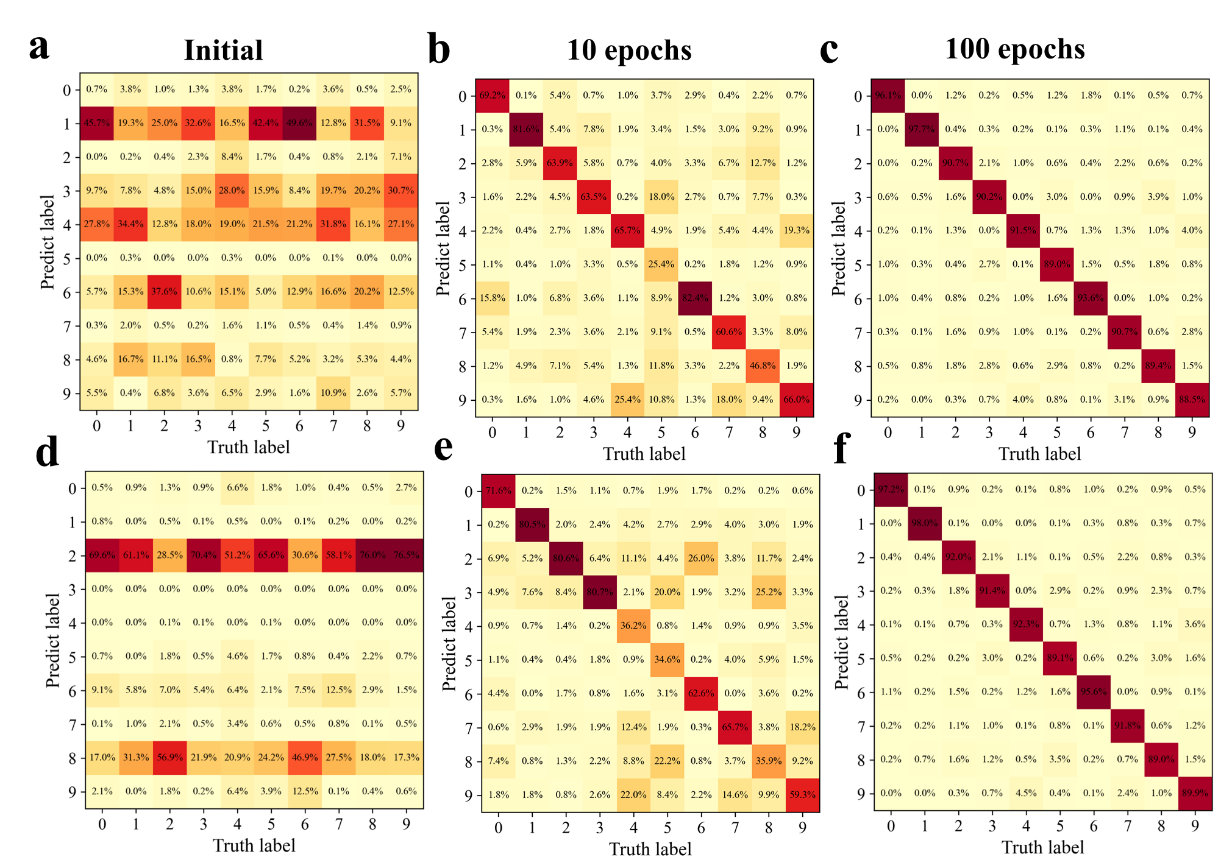


**Fig. S20.** Comparison of image processing with noise at 0.1. **a, b, c** Confusion matrix of training results under initial state, 10 and 100 epochs without NVS; **d, e, f** Confusion matrix of training results under initial state, 10 and 100 epochs with NVS.


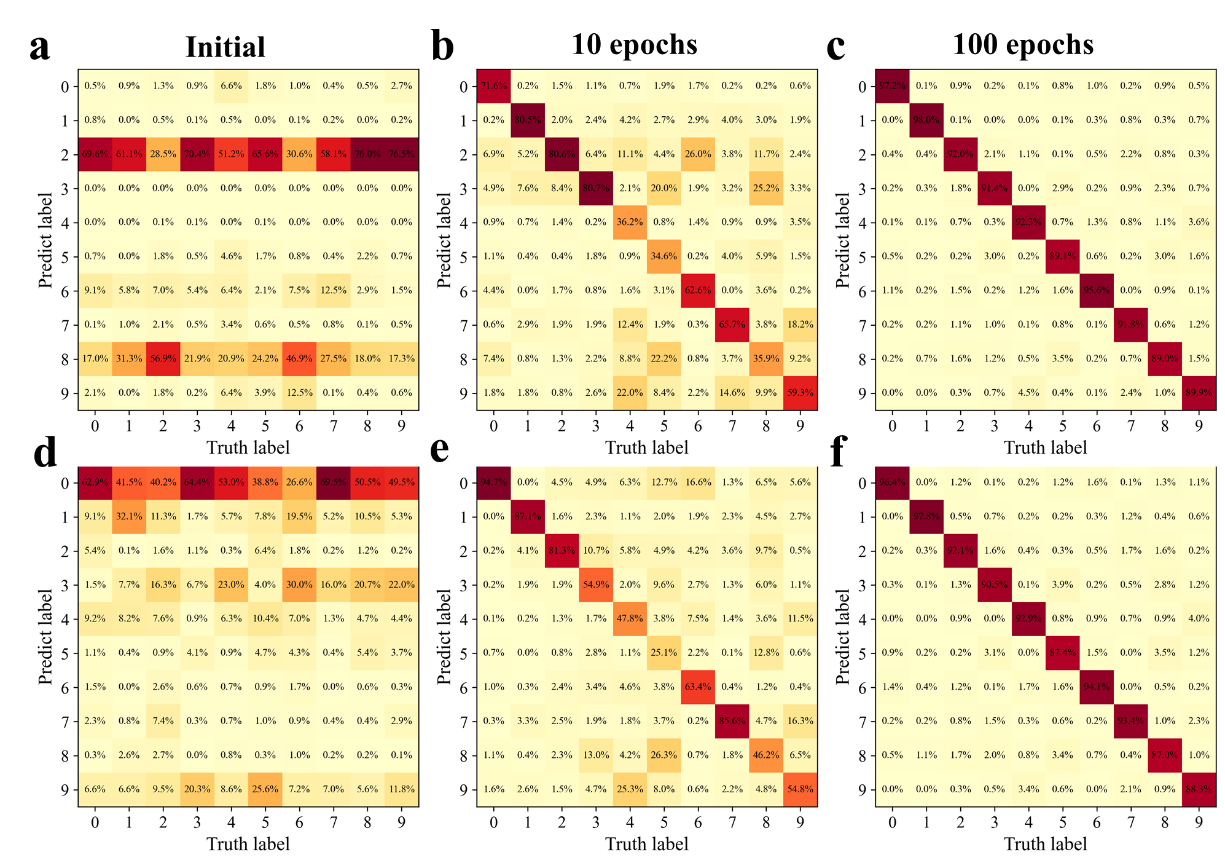


**Fig. S21.** Comparison of image processing with noise at 0.2. **a, b, c** Confusion matrix of training results under initial state, 10 and 100 epochs without NVS; **d, e, f** Confusion matrix of training results under initial state, 10 and 100 epochs with NVS.


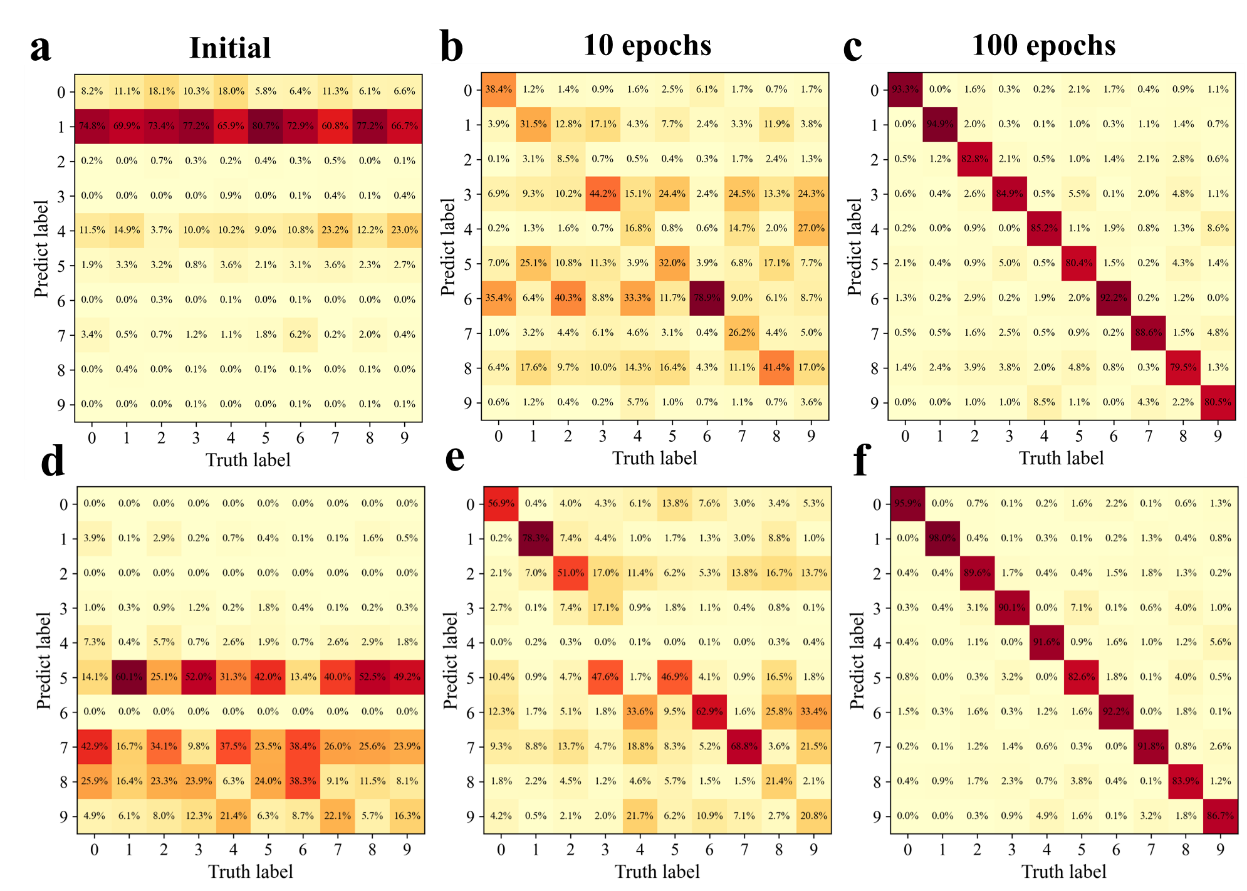


**Fig. S22.** Comparison of image processing with noise at 0.3. **a, b, c** Confusion matrix of training results under initial state, 10 and 100 epochs without NVS; **d, e, f** Confusion matrix of training results under initial state, 10 and 100 epochs with NVS.


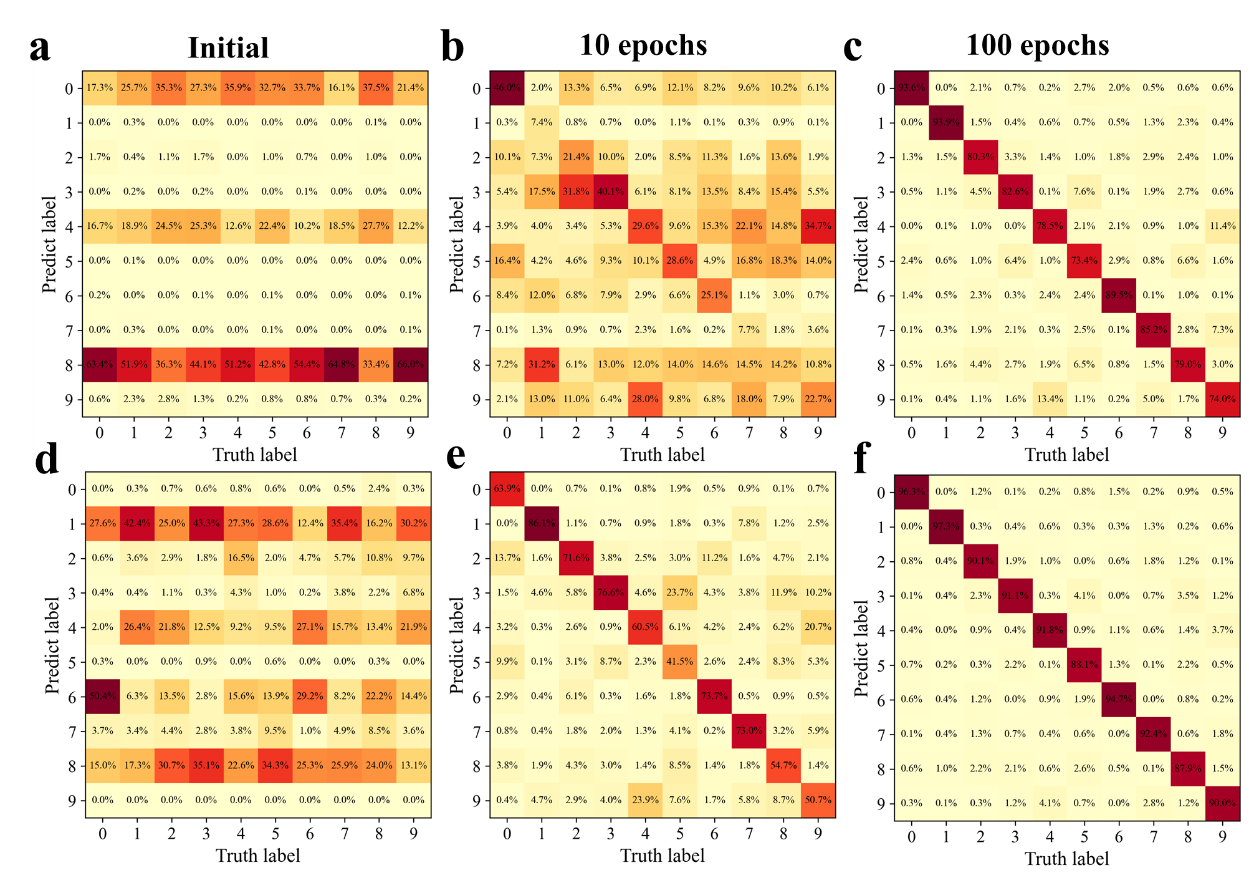


**Fig. S23.** Comparison of image processing with noise at 0.4. **a, b, c** Confusion matrix of training results under initial state, 10 and 100 epochs without NVS; **d, e, f** Confusion matrix of training results under initial state, 10 and 100 epochs with NVS.

**
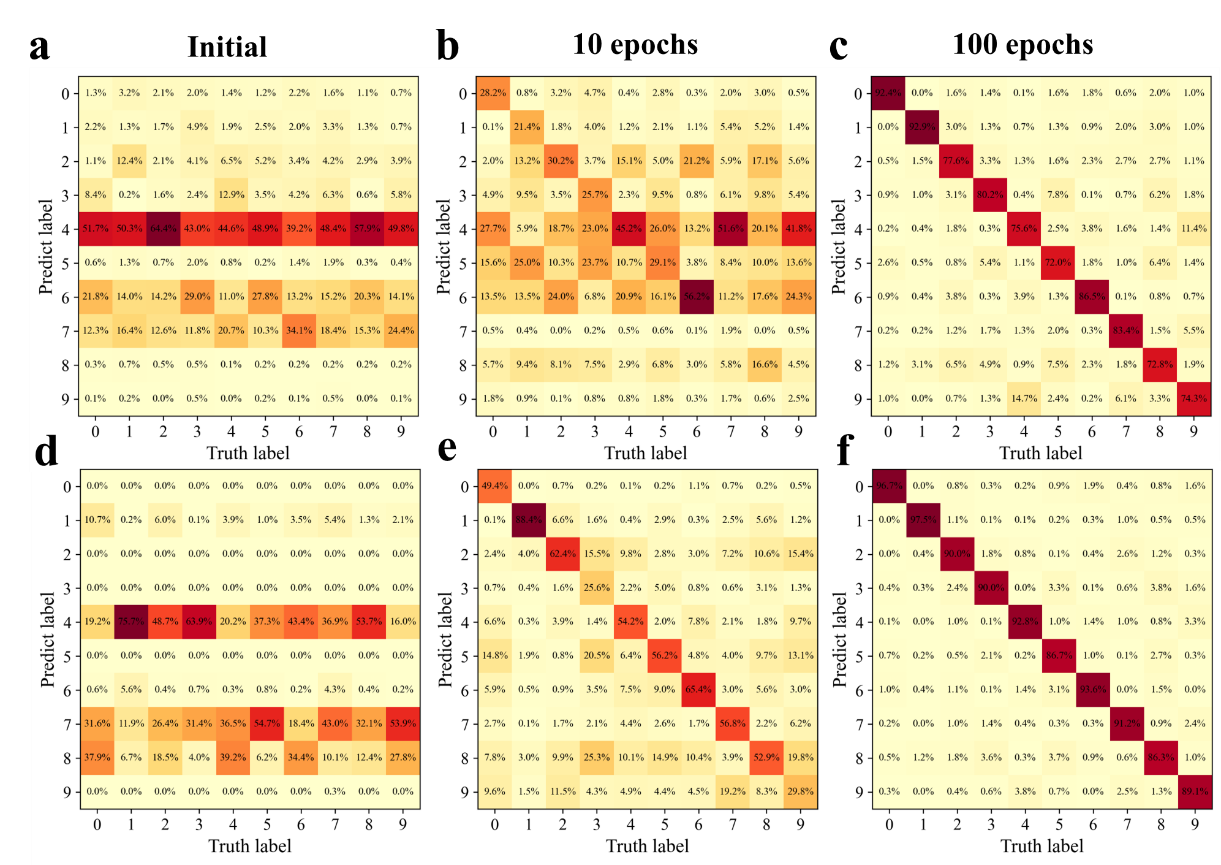
**

**Fig. S24.** Comparison of image processing with noise at 0.5. **a, b, c** Confusion matrix of training results under initial state, 10 and 100 epochs without NVS; **d, e, f** Confusion matrix of training results under initial state, 10 and 100 epochs with NVS.


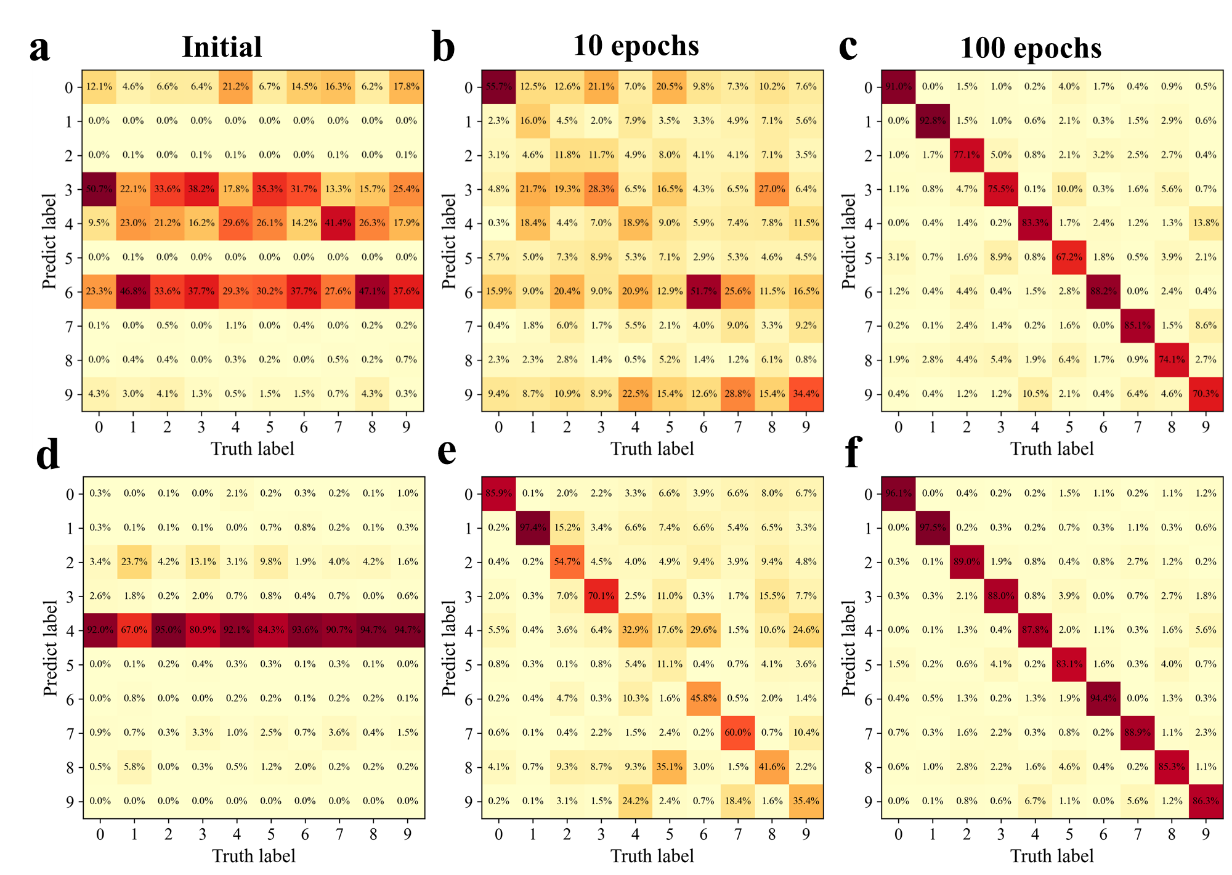


**Fig. S25.** Comparison of image processing with noise at 0.6. **a, b, c** Confusion matrix of training results under initial state, 10 and 100 epochs without NVS; **d, e, f** Confusion matrix of training results under initial state, 10 and 100 epochs with NVS.


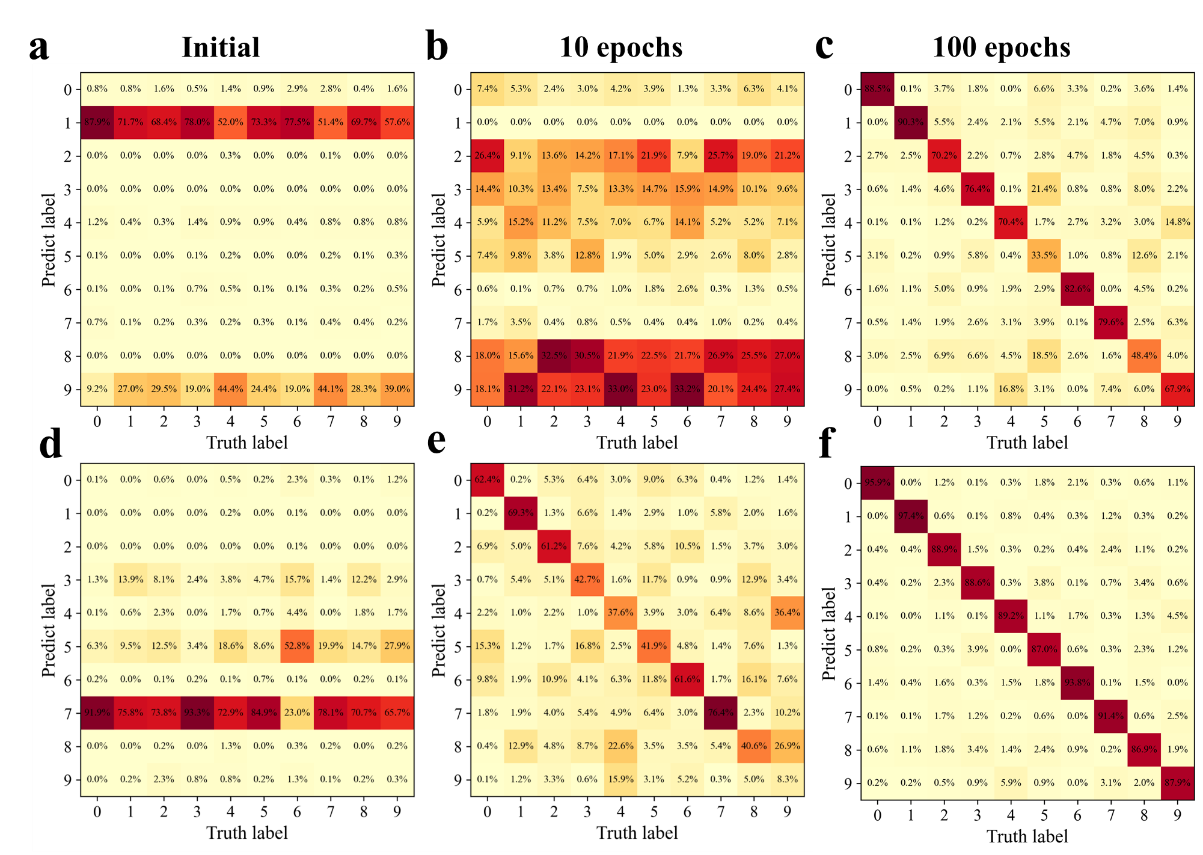


**Fig. S26.** Comparison of image processing with noise at 0.7. **a, b, c** Confusion matrix of training results under initial state, 10 and 100 epochs without NVS; **d, e, f** Confusion matrix of training results under initial state, 10 and 100 epochs with NVS.


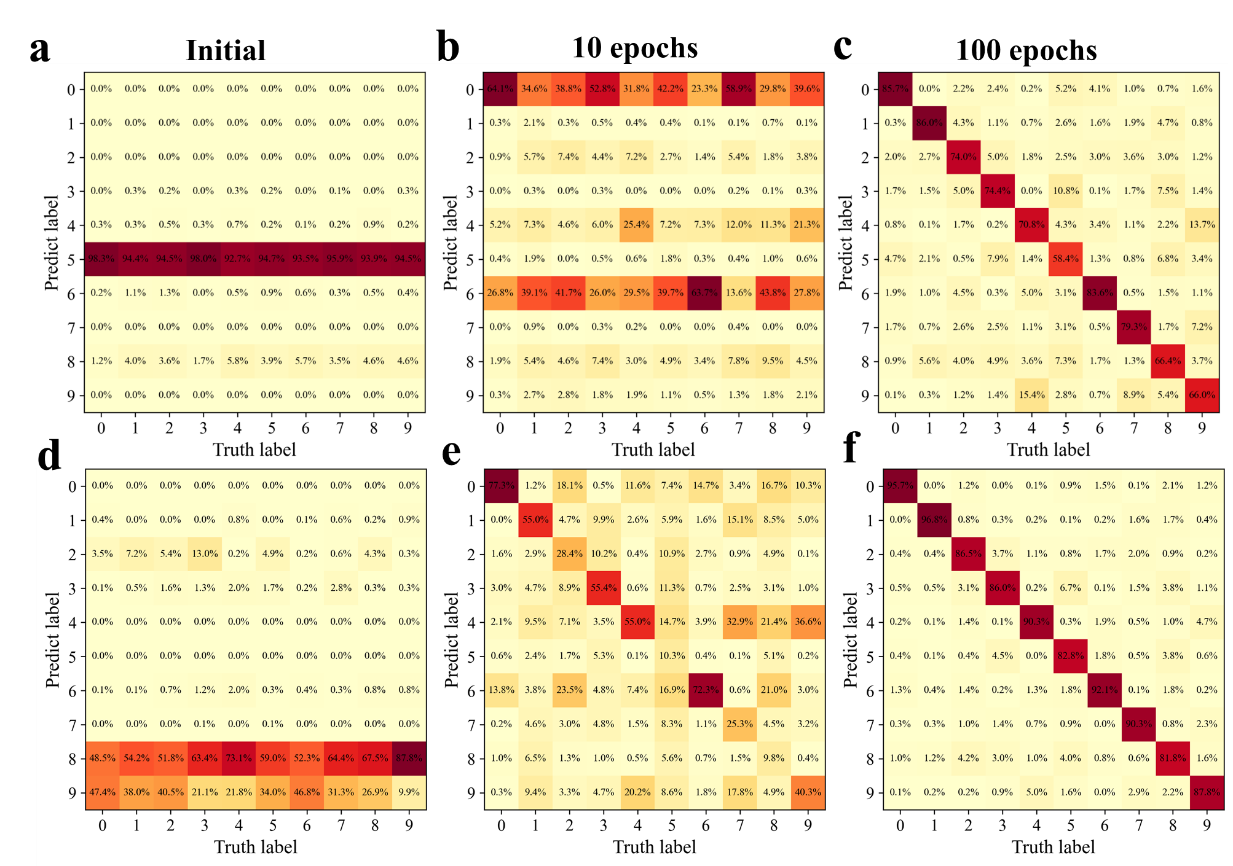


**Fig. S27.** Comparison of image processing with noise at 0.8. **a, b, c** Confusion matrix of training results under initial state, 10 and 100 epochs without NVS; **d, e, f** Confusion matrix of training results under initial state, 10 and 100 epochs with NVS.

Compared with the mixed matrix of recognition rate of 0, 10 and 100 training times, we found that the image recognition accuracy after NVS processing was better, the recognition rate was higher, and the training frequency was lower. It is proved that the device is more helpful to improve the efficiency of image recognition after NVS processing, and it is also a future vision system construction.


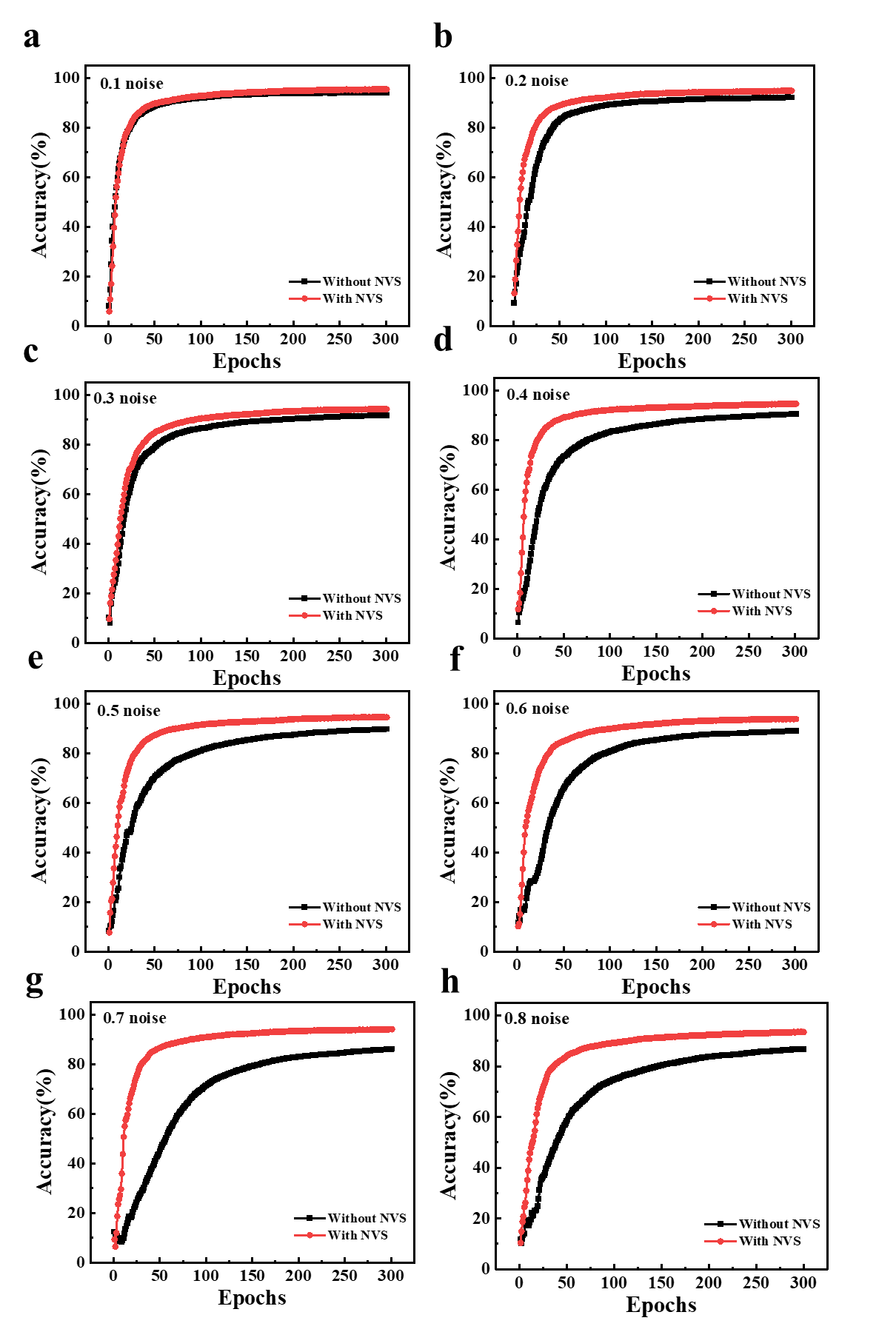


**Fig. S28.** Recognition accuracy of the training images without and with the image preprocessing based on as-prepared NVS under various noise levels. a-h 0.1~0.8.

**

**

**Fig. S29.** ΔRA (30 epochs) as a function of background noise level. The ΔRA is define the difference of recognition accuracy between with and without NVS ^4^. It can be seen that ΔRA increased as the noise level raised, suggesting the excellent denoising effect of the PRM devise for high noise. It is further proved that the NVS preprocessing based on PRM device can effectively improve the recognition rate of image recognition.





**Fig. S30.** The relationship between recognition accuracy and Δepoch under various noise levels. The Δepoch is defined the difference between the epochs required to achieve the same recognition accuracy without and with the NVS ^4^. One can see that the Δepoch increased from 3 to 185 with raising the noise level from 0.1 to 0.8 at the recognition accuracy of 85%. This result suggests that as the background noise level increases, the NVS becomes more effective in denoising and image recognition.

**Table S1** Published work on light energy density comparison.

| Device structure | Optical density | Published journal |
| --- | --- | --- |
| Ta/TaO*_x_*/NbO*_x_*/W | 50 ~ 80 mW/cm^2^ | Nat. Electron.^5^ |
| Au/WSe_2_/Al_2_O_3_/HfO_2_/Al_2_O_3_/Au | 50 mW/cm^2^ | Nat. Electron.^6^ |
| ITO/Ta_2_O_5_/Ag/IGZO/ITO | 0.03-0.5 nW/μm^2^ | Nat. Commun. ^7^ |
| ITO/ZnO/P1:PC_71_BM/MoO_3_/Ag | 100 mW/cm^2^ | Angew. Chem. Int. Ed.^8^ |
| Au/BaSnO_3_/MaO/Au | 70 mW/cm^2^ | Nat. Commun.^9^ |
| Au/PdSe_2_/MoTe_2_/Au | 0.23 nW/μm^2^ | Nat. Electron.^10^ |
| Pd/ZnO/Graphene/SiO_2_/Si | 0.228 mW/mm^2^ (0.228 nW/μm^2^) | **This work** |

**Table S2** Recently Reported Reconfigurable Memristor.

| Device structure | Switching method | Volatile threshold switching memristor (VTSM) | | Nonvolatile  memristor (NVM) | | Resistance value of VTSM and NVM |
| --- | --- | --- | --- | --- | --- | --- |
|  |  | Voltage (set) | Current | Voltage (set) | Current |  |
| Ag/MoS_2_/HfAlOx/CNT^11^ | Compliance current（CC） | 3V | 100nA | 1~3V | 1μA~1mA | Nonuniform |
| Ag/CsPbBr_3_NCs/pTPD PEDOT:PSS/Ag^12^ | CC | ~0.5V | 10^-6^A | ~1.5V | 10^-3^A | Nonuniform |
| Ag/TaO_x_/TaO_y_/TaO_x_/Ag^13^ | CC | 0.16V­­–0.2V | 100nA--100μA | 0.16V | 500μA | Nonuniform |
| Ag/CuInSe_2_/Mo^14^ | CC | 1V | 10^-5^A | 1V | 10^-3^A | Nonuniform |
| Ag/AIZS/Cs_3_Cu_2_Cl_5_/W^15^ | CC | ~0.45V–0.75V | 100μA | ~1.5V | 300μA | Nonuniform |
| V/VO*x*/HfWOx/Pt^16^ | Filament in the RS layer | ~1.1V–1.3V | 10^-3^A | ~1.8V–2.5V | 10^-3^A | Nonuniform |
| Ag/HfO_2_/NiO/Pt^17^ | CC | ~0.3V | 100μA | 0.2V–0.4V | 10mA | Nonuniform |
| Cu_x_Te_1-x_/HfO_2_/Pt^18^ | CC | ~3V | 10 nA | ~1.25V | 100 nA | Nonuniform |
| Au/Ag NW–P(VDF-HFP)/Au^19^ | CC | ~0.3V | 10^-4^A | ~0.8V | 10^-3^A | Nonuniform |
| Pt/TiN/Ge_3_Se_7_/TiN/Cu-GeSe/TiN Ge_3_Se_7_/Wu^20^ | CC | ~0.65V | 10^-6^A | ~0.85V | 10^-4^A | Nonuniform |
| Ag/HfO_2_/Pt^21^ | CC | ~0.4V | 100μA | ~0.2V | 1mA | Nonuniform |
| Ag/milk/Pt/SiO_2_/Si | CC | ~0.9V | 1μA | ~0.75V | 100μA | Nonuniform |
| Ag/InP/ZnS/ITO^22^ | CC and UV light | ~0.75V | 0.1mA | ~1.25–1.75V | 0.1mA | Nonuniform |
| Ag/ZrO_2_/Pt^23^ | CC | ~0.3–0.45V | 10^-5^A | ~ 0.4V | 10^-4^A | Nonuniform |
| Au/Ag/PMMA/OIHP:Ag/ITO^24^ | CC | ~0.25V | 10^-7^A | ~0.25V | 10^-6^A | Nonuniform |
| Ag/CIPS/Au^25^ | CC | ~0.4V | 10^-5^A | ~0.5V | 10^-3^A | Nonuniform |
| Ag/SiO_2_/Pt^26^ | CC | ~0.9V | 10^-5^A | ~0.8V | 10^-4^A | Nonuniform |
| Ag/SiO_2_/ITO^27^ | CC | ~0.3V | 10^-7^A | ~1.5V | 10^-2^A | Nonuniform |
| Graphene/ZnO/Pd (**This work**) | Light on and off | 2V | 66μA | 2V | 66μA | Uniform |

**Reference**

1 Li, B., Liu, T., Wang, Y. & Wang, Z. ZnO/graphene-oxide nanocomposite with remarkably enhanced visible-light-driven photocatalytic performance. *J. Colloid Interface Sci.* **377**, 114-121, (2012).

2 Yu, J., Suleiman, A. A., Zheng, Z., Zhou, X. & Zhai, T. Giant‐enhanced sns_2_ photodetectors with broadband response through oxygen plasma treatment. *Adv. Functi. Mater.* **30**, 2001650, (2020).

3 Tan, H. *et al.* An optoelectronic resistive switching memory with integrated demodulating and arithmetic functions. *Adv. Mater.* **27**, 2797-2803, (2015).

4 Feng, S. *et al.* Dual‐mode conversion of photodetector and neuromorphic vision sensor via bias voltage regulation on a single device. *Adva. Mater.* **35**, 2308090, (2023).

5 Dang, B. *et al.* Reconfigurable in-sensor processing based on a multi-phototransistor–one-memristor array. *Nat. Electron.* **7**, 991-1003, (2024).

6 Zhou, Y. *et al.* Computational event-driven vision sensors for in-sensor spiking neural networks. *Nat. Electron.* **6**, 870-878, (2023).

7 Wang, X. *et al.* Vertically integrated spiking cone photoreceptor arrays for color perception. *Nat. Commun.* **14**, 3444, (2023).

8 Wang, S. *et al.* Retina‐inspired organic photonic synapses for selective detection of swir light. **62**, e202213733, (2023).

9 Li, P. *et al.* Reconfigurable optoelectronic transistors for multimodal recognition. *Nat. Commun.* **15**, 3257, (2024).

10 Pi, L. *et al.* Broadband convolutional processing using band-alignment-tunable heterostructures. *Nat. Electron.* **5**, 248-254, (2022).

11 Wang, T. *et al.* Reconfigurable neuromorphic memristor network for ultralow-power smart textile electronics. *Nat. Commun.* **13**, 7432, (2022).

12 John, R. A. *et al.* Reconfigurable halide perovskite nanocrystal memristors for neuromorphic computing. *Nat. Commun.* **13**, 2074, (2022).

13 Sun, Y. *et al.* Performance‐enhancing selector via symmetrical multilayer design. *Adv. Funct. Mater.* **29**, 1808376, (2019).

14 Guo, T. *et al.* Versatile memristor for memory and neuromorphic computing. *Nanoscale Horiz.* **7**, 299-310, (2022).

15 He, N. *et al.* Multifunctional Ag–In–Zn–S/Cs_3_Cu_2_Cl_5_‐based memristors with coexistence of non‐volatile memory and volatile threshold switching behaviors for neuroinspired computing. *Adv. Electron. Mater.* **9**, 2201038, (2022).

16 Fu, Y. *et al.* Reconfigurable synaptic and neuronal functions in a V/VO*_x_*/HfWo*_x_*/pt memristor for nonpolar spiking convolutional neural network. *Adv. Funct. Mater.* **32**, 2111996, (2022).

17 Chen, J. *et al.* Reconfigurable Ag/HfO_2_/NiO/Pt memristors with stable synchronous synaptic and neuronal functions for renewable homogeneous neuromorphic computing system. *Nano Lette.* **24**, 5371-5378, (2024).

18 Woo, K. S. *et al.* Tunable stochastic memristors for energy-efficient encryption and computing. *Nat. Commun.* **15**, 3245, (2024).

19 Wang, D. *et al.* All‐flexible artificial reflex arc based on threshold‐switching memristor. *Adv. Funct. Mater.* **32**, 2200241, (2022).

20 Kim, H. J. *et al.* Super‐linear‐threshold‐switching selector with multiple jar‐shaped cu‐filaments in the amorphous Ge_3_Se_7_ resistive switching layer in a cross‐point synaptic memristor array. *Adv. Mater.* **34**, 2203643, (2022).

21 Abbas, H. *et al.* The coexistence of threshold and memory switching characteristics of ald HfO_2_ memristor synaptic arrays for energy-efficient neuromorphic computing. *Nanoscale* **12**, 14120-14134, (2020).

22 Wang, J. *et al.* Optically modulated threshold switching in core–shell quantum dot based memristive device. *Adv. Funct. Mater.* **30**, 1909114, (2020).

23 Yang, J. H., Mao, S. C., Chen, K. T. & Chen, J. S. Emulating nociceptive receptor and lif neuron behavior via zrox‐based threshold switching memristor. *Adv. Electron. Mater.* **9**, 2201006, (2022).

24 Tang, L. *et al.* A low power flexible halide perovskite-based threshold switching memristor as an artificial nociceptor. *J. Mater. Chem. C* **12**, 3622-3631, (2024).

25 Liu, Y. *et al.* Versatile memristor implemented in van der waals CuInP_2_S_6_. *Nano Res.* **16**, 10191-10197, (2023).

26 Sun, Y. *et al.* Design of a controllable redox‐diffusive threshold switching memristor. *Adv. Electron. Mater.* **6**, 2000695, (2020).

27 Li, Z. *et al.* On bidirectional transition between threshold and bipolar switching in Ag/SiO_2_/ITO memristors. *IEEE Trans. Nanotechnol.* **23**, 771 - 777, (2024).
